# Developing Metal–Polyphenol Network‐Based Single‐Protein Particle Encapsulation System for Sustained Release of Whey Protein to Mitigate Sarcopenia

**DOI:** 10.1002/advs.77951

**Published:** 2026-09-27

**Authors:** Yafei Zhang, Yuning Zhang, Yongjian Ai, Yao Lu, Heng Quan, Zhen Zhang, Yuqi Wang, Yujia Luo, Xiaoxu Zhang, Yingying Lin, Sijia Song, Huiyuan Guo

**Affiliations:** ^1^ College of Food Science and Nutritional Engineering China Agricultural University Beijing China; ^2^ MOE Key Laboratory of Precision Nutrition and Food Quality, Department of Nutrition and Health China Agricultural University Beijing China

**Keywords:** metal polyphenol networks, muscular atrophy, saccharide, sarcopenia, self‐assembly, single‐protein particle encapsulation system, whey protein

## Abstract

Sarcopenia has emerged as a major health challenge in the aging population. Whey protein (WP)—rich in branched‐chain amino acids—is an important nutritional supplement for mitigating sarcopenia. However, the rapid digestion of WP limits its therapeutic efficacy. Developing a sustained‐release system for WP may enhance its effectiveness in mitigating sarcopenia. Herein, a novel “single‐protein particle encapsulation” (SPPE) system is developed to protect WP and control its release. Specifically, the “molecular glue” metal–polyphenol networks (MPNs) are assembled onto the surface of individual polymerized WP isolate (PWPI) particles as an intermediate layer and bridged with the saccharide hyaluronic acid (HA) to construct PWPI@MPNs/HA. The results showed that PWPI@MPNs/HA precisely regulates the release rate of PWPI in the gastrointestinal tract. In a murine sarcopenia model, PWPI@MPNs25/HA significantly improves lean body content, grip strength, grid hanging time, exhaustion time, and exhaustion distance. The underlying molecular mechanism involves activation of the PI3K/AKT/mTOR signaling pathway and suppression of the muscle atrophy factors *MuRF‐1* and *Atrogin‐1* in the ubiquitin–proteasome system via PWPI@MPNs/HA. The SPPE system developed in this study may have broad future applications in protein protection and delivery.

## Graphical abstract

Sarcopenia has emerged as a major health challenge in the aging population. Developing a sustained‐release system for whey protein may enhance its effectiveness in mitigating sarcopenia. Herein, a novel “single‐protein particle encapsulation” (SPPE) system is developed to protect whey protein and control its release. The SPPE system may have broad future applications in protein protection and delivery.

## 1 Introduction

Population aging is a global phenomenon associated with an increasing prevalence of musculoskeletal and age‐related conditions, including osteoporosis, cognitive impairment, and sarcopenia [1, 2]. Sarcopenia can lead to disability, loss of independent living, and physical weakness [3]. Therefore, sarcopenia is a major public health concern that significantly reduces the quality of life of older adults [4]. Nutritional intervention is a promising and well‐accepted strategy for managing sarcopenia, with the potential to enhance patient well‐being [1]. Currently, whey protein (WP) is the primary protein used in nutritional interventions for sarcopenia [5]. Compared with other dietary proteins, WP contains high levels of essential amino acids (EAAs) and branched‐chain amino acids (BCAAs) [6]. BCAAs account for 35% of the amino acids (AAs) in skeletal muscle proteins and play an essential role in stimulating muscle protein synthesis (MPS) [7]. Since the human body cannot synthesize BCAAs, they must be obtained from dietary sources. Therefore, WP effectively increases the plasma concentrations of EAAs and BCAAs, promoting the initiation of MPS. During MPS, substantial amounts of non‐essential amino acids (NEAAs) are also required. WP supplies abundant NEAAs as building blocks for MPS. The loss of muscle protein is due to the rate of muscle protein breakdown (MPB) exceeding that of MPS. Skeletal muscle proteins undergo a daily turnover rate of approximately 2%, reflecting the combined rates of MPB and MPS. Continuous AA availability is essential for sustaining MPS [8]. However, WP is classified as a “fast‐digesting protein,” and plasma AA concentrations typically peak approximately 1 h after ingestion. Most AAs entering the plasma at high concentrations over a short period are metabolized to urea rather than incorporated into muscle protein. Therefore, developing a sustained‐release system for WP to enable its timely and sustained release is important for the nutritional management of sarcopenia. Currently, most studies on sustained‐release WP for MPS use enzymatic cross‐linking to produce “casein‐like” WP in a colloidal state [9]. However, this approach has some limitations. First, colloidal WP may cause swallowing difficulty and gastrointestinal discomfort in older adults [10]. Second, the digestion rate of the gel is difficult to control. For instance, soft and hard WP gels are hydrolyzed after 240 min of digestion [11]. Therefore, new strategies are urgently needed to achieve the controllable release of WP.

In recent years, multivalent self‐assembled coatings formed through chelation between metal ions and polyphenols have attracted considerable attention [12, 13]. These coatings—known as metal–polyphenol networks (MPNs)—can be rapidly assembled on various substrate materials with different surface topologies and sizes within a few minutes [14, 15]. As the applications of MPNs continue to expand, these network structures have been found to protect encapsulated substances from adverse external conditions, such as ultraviolet radiation and enzymatic degradation, while also exhibiting targeting properties [12, 16]. Whey protein isolate (WPI) contains abundant functional groups, including amino, hydroxyl, and carboxyl groups, which may facilitate MPNs assembly on its surface. However, MPNs exhibit pH‐responsive behavior, allowing rapid assembly under neutral or alkaline conditions and disassembly under acidic conditions [17]. MPNs can function as a “molecular glue.” Therefore, MPNs are hypothesized to serve as an intermediate coating on the surface of WPI and bind to saccharides, forming a double‐layer nanoshell structure through layer‐by‐layer self‐assembly. Saccharides with resistance to pH changes and enzymatic digestion, similar to a seed coat, may protect WPI and regulate its release. In this study, proanthocyanidins (PC), calcium ions (Ca^2+^), and hyaluronic acid (HA) were selected as the model polyphenol, metal ion, and saccharide, respectively.

**SCHEME 1:**
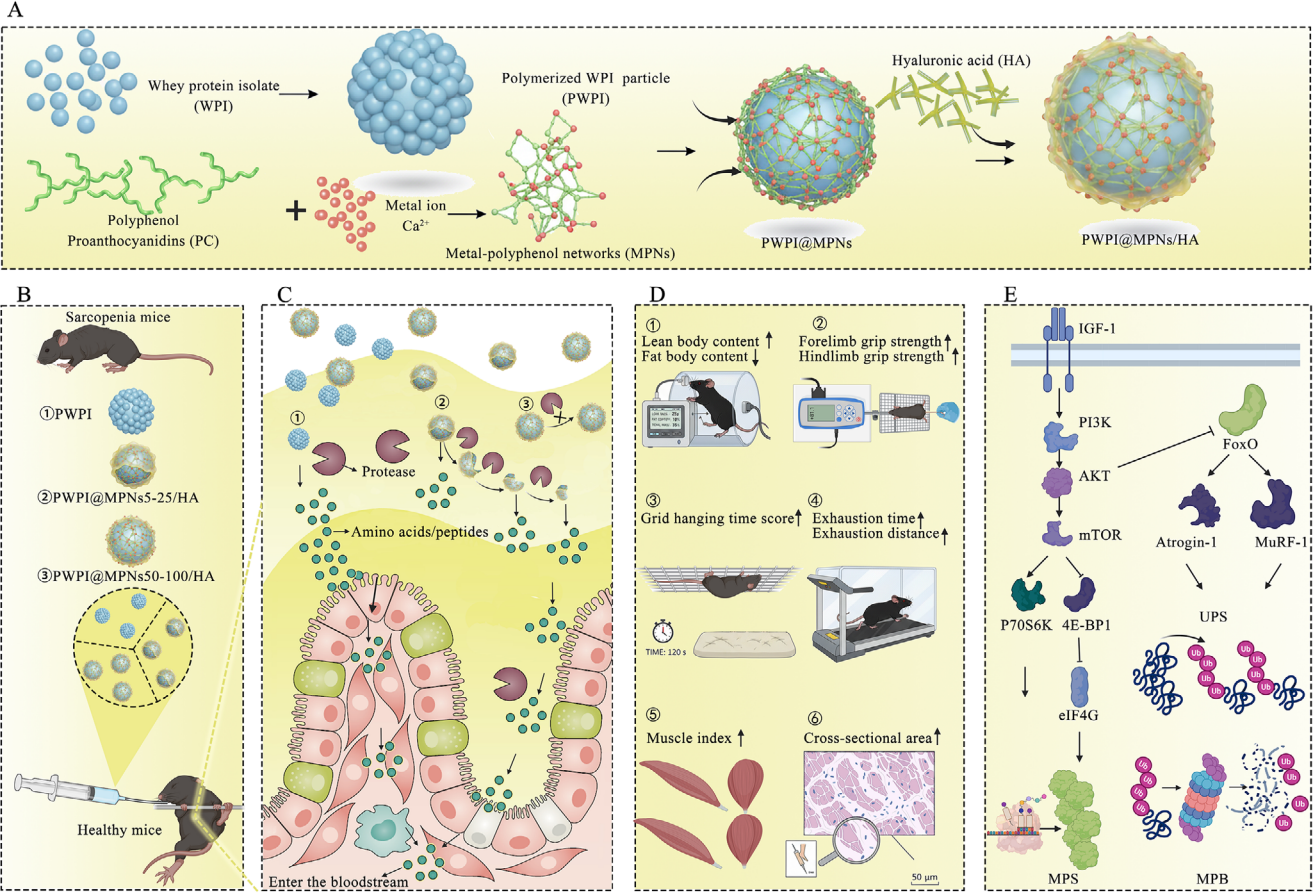
(A) Schematic diagram of the preparation process of PWPI@MPNs/HA; (B) The steps of in vitro digestion; (C, D) Investigation of the sustained‐release effect and the therapeutic effect of PWPI@MPNs/HA on sarcopenia in a mouse model; (E) Molecular mechanism of PWPI@MPNs/HA in alleviating sarcopenia.

Inspired by the structure of a seed, a novel “single‐protein particle encapsulation (SPPE)” system was developed. First, WPI was converted into polymerized WPI (PWPI) particles to reduce MPN usage and facilitate encapsulation (Scheme 1A). Then, MPNs were coated onto the surface of individual PWPI particles through coordination‐driven self‐assembly to produce PWPI@MPNs. Finally, the “seed coat” material, HA, was self‐assembled onto the PWPI@MPNs surface to form PWPI@MPNs/HA. The formation mechanism of PWPI@MPNs/HA was then analyzed, and the digestion rate of PWPI was evaluated using an in vitro digestion model. A mouse model of sarcopenia was used to verify the therapeutic efficacy of PWPI@MPNs/HA (Scheme 1B–D), and its underlying molecular mechanism was further explored (Scheme 1E).

## 2 Results

### 2.1 Preparation and Polymerization Mechanism Analysis of Polymerized Whey Protein Isolate

Converting WPI into PWPI reduces the amount of MPNs and HA required for the outer “seed coat.” Therefore, heat‐reduced polymerization under the isoelectric point (pI) of WPI was employed. First, the pI of WP was determined to be 4.5 (Figure S1). WPI was then heated at pH 4.5 and 90°C for 0–40 min. In the early stage of heating, with the extension of the heating time, the PWPI particles gradually became larger. After 20 min of heating, the particle size remained unchanged (Figure S2), and the degree of polymerization reached a plateau. In addition, turbidity did not increase (Figure S3), and solubility did not decrease after 20 min of heating (Figure S4). These results were consistent with the digital images (Figure S5). Therefore, the optimal conditions for PWPI preparation were pH 4.5, 90°C, and 20 min of heating. Next, the polymerization mechanism of WPI was analyzed. During heating, the surface hydrophobicity of WPI (Figure S6) and the content of surface free thiol groups (Figure S7) gradually increased, indicating structural changes that exposed internal hydrophobic groups. The fluorescence intensity of WPI also gradually increased during heating (Figure S8 and Table S1). After heating for ≥ 10 min, λ_max_ exceeded 330 nm and shifted toward longer wavelengths, indicating a redshift and exposure of internal hydrophobic tryptophan residues to a hydrophilic environment. The FT‐IR spectrum (Figure S9) showed an O─H stretching vibration at 3293cm^−1^. As the heating time increased, a slight blue shift was observed, indicating weakened hydrogen‐bond (H‐bond) interactions. Analysis of the amide I band (1700–1600 cm^−1^) using curve fitting, Fourier self‐deconvolution, and peak‐fitting methods showed a gradual decrease in the β‐sheet content, while the α‐helix content remained essentially unchanged. The β‐turn content gradually decreased, whereas the random coil content increased, indicating a gradual transition of the PWPI structure from an ordered to a disordered state (Table S2). Overall, heating exposed the internal hydrophobic groups and disulfide bonds of WPI, promoted a structural transition from an ordered to a disordered state, and subsequently induced aggregation through hydrophobic interactions and disulfide bonds.

### 2.2 Preparation and Characterization of PWPI@MPNs/HA

**FIGURE 1:**
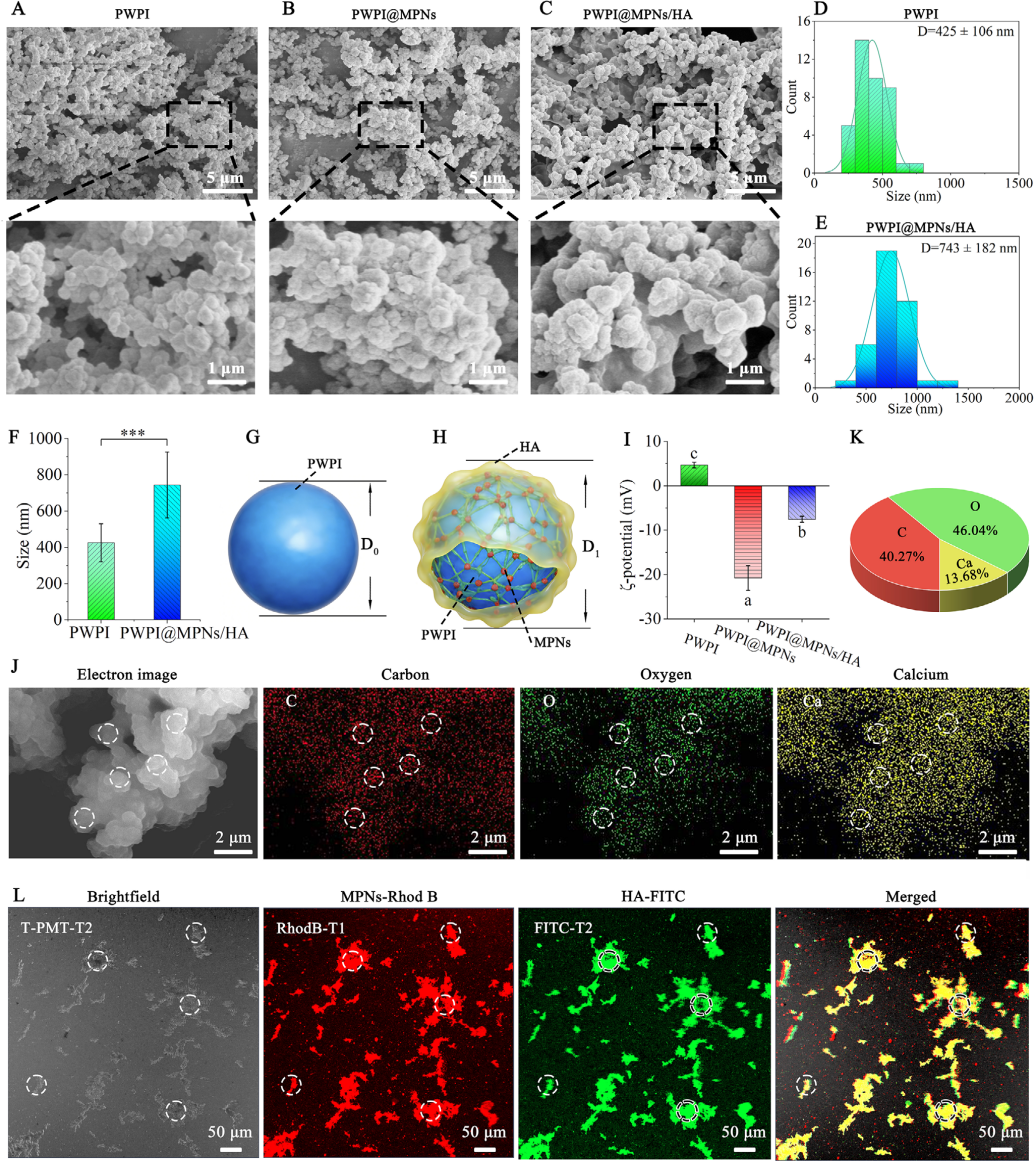
(A–C) The SEM image of the PWPI, PWPI@MPNs and PWPI@MPNs/HA; (D) PWPI particle size statistics; (E) PWPI@MPNs/HA particle size statistics; (F) PWPI and PWPI@MPNs/HA particle size comparison; (G, H) Schematic diagram of MPNs/HA thickness calculation; (I) The ζ‐potential of PWPI, PWPI@MPNs, and PWPI@MPNs/HA; (J) Observation of the positions of carbon, oxygen and calcium elements in PWPI@MPNs/HA by EDS; (K) Semi‐quantitative results of carbon, oxygen and calcium elements in PWPI@MPNs/HA by EDS; (L) The positions of MPNs‐RhB and HA‐FITC were observed using CLSM and co‐positioned with PWPI. Mean ± SE of *n* = 3 (at least) independent experiments. The “***” note as very significant difference with *p* < 0.001. Different lowercase letters indicated significant differences within groups (*p* < 0.05).

PWPI, PWPI@MPNs, and PWPI@MPNs/HA were subsequently prepared and characterized via scanning electron microscopy (SEM; Figure 1A–C). The SEM images showed that, although the WPs polymerized to form PWPI, individual WP microspheres remained distinguishable within the microstructure (Figure 1A). After MPN encapsulation, the PWPI particles became more compact, and the WP particles changed from spherical to irregular morphologies (Figure 1B). After HA encapsulation, the PWPI particles increased significantly in size (Figure 1C). The particle sizes of PWPI and PWPI@MPNs/HA were measured using ImageJ software, whereas PWPI@MPNs was excluded because of its irregular surface morphology. The results showed that the individual WP microspheres in PWPI had a particle size of 425 ± 106 nm (Figure 1D). The polymerized WP particles formed by moderate heating had a diameter of 50.4 µm (Figure S2). Aggregation of these WP microspheres into larger particles reduced the specific surface area and decreased the amount of the outer layer MPN/HA coating required. The microspheres in PWPI@MPNs/HA had a particle size of 743 ± 182 nm (Figure 1E), which differed significantly from that of the microspheres in PWPI (Figure 1F). Figure 1G,H shows the proposed encapsulation of MPNs/HA, and the thickness of the MPN/HA coating was calculated to be approximately 159 nm. The ζ‐potential of PWPI was 4.66 mV (Figure 1I). After MPNs bound to the PWPI surface, the ζ‐potential decreased to −20.80 mV. This reversal in ζ‐potential may be attributed to the abundant phenolic hydroxyl groups in the polyphenols, which carry a strong negative charge. After HA was assembled as the outermost layer, the ζ‐potential of PWPI@MPNs/HA increased significantly to −7.56 mV, due to the shielding effect of HA. The Ca^2+^ content and distribution in PWPI@MPNs/HA were analyzed using an energy‐dispersive spectrometer (EDS). The EDS images showed that Ca^2+^ was uniformly distributed on the PWPI surface (Figure 1J), with a semiquantitative Ca^2+^ content of 13.68% (Figure 1K). Rhodamine B and FITC were then used to label MPNs and HA, respectively. Confocal laser scanning microscopy co‐localization analysis showed that MPNs and HA colocalized with PWPI (Figure 1L). Overall, these results showed that MPNs/HA were successfully encapsulated on the PWPI surface.

### 2.3 Analysis of the PWPI@MPNs/HA Self‐Assembly Mechanism

**FIGURE 2:**
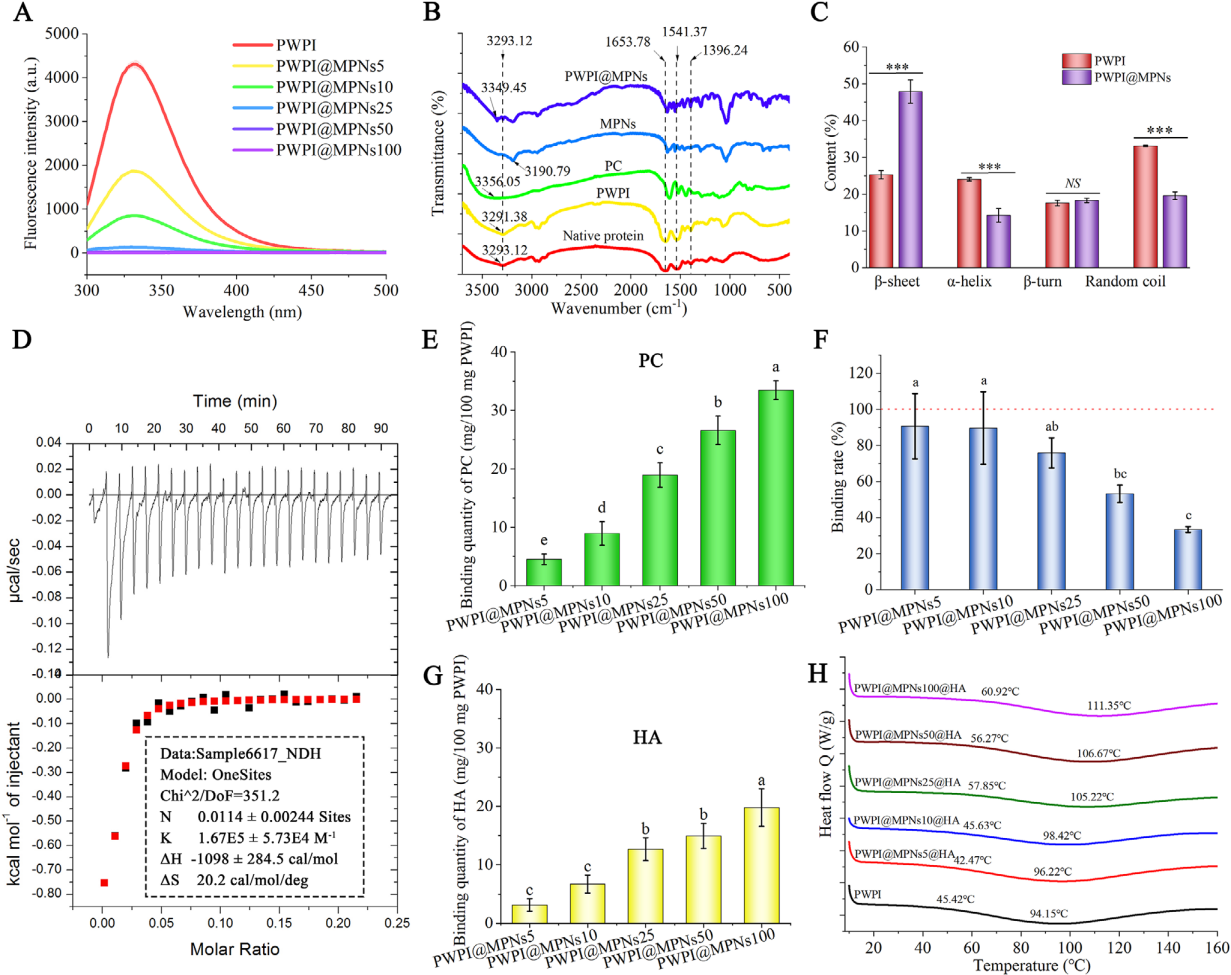
Mechanism analysis of MPNs and HA adsorption on PWPI, including (A) fluorescence spectra after different proportions of MPNs binding to PWPI; (B) FT‐IR results of WPI, PWPI, PC, MPNs, and PWPI@MPNs; (C) the changes in the contents of β‐sheet, α‐helix, β‐turn, and Random coil after MPNs binding to PWPI; (D) The change in energy during isothermal titration between MPNs and HA; (E) The amount of PC that could be combined with every 100 mg of PWPI; (F) The binding efficiency of the added MPNs with PWPI; (G) the amount of HA that could be adsorbed per 100 mg of PWPI in the PWPI@MPNs system; (H) The DCS curve of PWPI and PWPI@MPNs5/HA‐PWPI@MPNs100/HA. Mean ± SE of *n* = 3 (at least) independent experiments. The “***” note indicates a very significant difference with *p* < 0.001. Different lowercase letters indicate significant differences within groups (*p* < 0.05).

The self‐assembly mechanism of MPNs and HA on the PWPI surface was then analyzed. Fluorescence spectroscopy showed that the maximum fluorescence intensity (a.u._max_) gradually decreased from PWPI to PWPI@MPNs5–PWPI@MPNs100 (Figure 2A; Table S3). This result showed reduced exposure of hydrophobic fluorescent groups and decreased surface hydrophobicity of the protein. Therefore, WPI and MPNs likely interacted through hydrophobic interactions. In the PWPI@MPNs25 group, the protein fluorescence was almost completely quenched, with a fluorescence quenching efficiency of 96.91 ± 0.16% (Table S3). The calculated binding constant (*K_a_*) and the number of binding sites (*n*) between PWPI and MPNs were 6.7 × 10^8^ L·mol^−1^ and 1.96, respectively. *K_a_* reflects the binding affinity between proteins and ligands, with higher *K_a_* values indicating stronger binding [18]. Liang et al. report a *K_a_* of 1.02 × 10^6^ L·mol^−1^ for the interaction between pterostilbene and WP, indicating that PWPI and MPNs exhibited stronger binding affinity [18]. The quenching rate constant (*K_q_*) and Stern–Volmer quenching constant (*K_SV_*) were 1.54 × 10^13^ L·mol^−1^·s^−1^ and 1.54 × 10^5^ L·mol^−1^, respectively. The maximum dynamic quenching constant (*K_q_*) was 2.0 × 10^10^ L·mol^−1^·s^−1^. Therefore, the fluorescence quenching mechanism of MPNs was static quenching rather than dynamic quenching [19]. Unheated WPI and PWPI exhibited O─H stretching peaks at 3293 and 3291 cm^−1^, respectively (Figure 2B). The O─H stretching peak of PC was significantly redshifted to 3356 cm^−1^ with a substantially larger peak area, likely because of the abundant hydroxyl groups in PC. The O─H stretching peak in MPNs was blueshifted to 3190.79 cm^−1^ with reduced peak intensity, indicating that Ca^2+^ coordinated with the ─OH groups of PC and disrupted H‐bond interactions within PC. Compared with PWPI, the O─H stretching peak of PWPI@MPNs was redshifted to 3349 cm^−1^, indicating enhanced H‐bond interactions and suggesting that MPNs interacted with PWPI through H‐bonds. Furthermore, compared with PWPI, the amide I (1653 cm^−1^) and amide II (1541 cm^−1^) bands of PWPI@MPNs were significantly weakened, suggesting that the binding sites of PWPI may involve C═O and N─H groups [19]. In addition, after MPNs were combined with PWPI, the structure of PWPI changed from a disordered to a more ordered state (Figure 2C). Overall, MPNs may interact with PWPI through hydrophobic interactions and H‐bonds.

To analyze the potential interaction between MPNs and HA, isothermal titration calorimetry was performed (Figure 2D). According to the equation Δ*G* (Gibbs free energy) = Δ*H* (enthalpy change) − TΔ*S* (entropy change), the calculated Δ*G* was −1603 cal/mol. Since Δ*G* < 0, the interaction between MPNs and HA occurred spontaneously [20]. The binding constant *K* was 1.67 × 10^5^ M^−1^. In comparison, the reported *K* for pea protein and grape seed proanthocyanidins was 3.76 × 10^3^ M^−1^ [21], indicating a stronger binding affinity between MPNs and HA. Δ*H* = −1098 cal/mol; Since Δ*H* < 0, the interaction was enthalpy‐driven, suggesting that H‐bonds were the dominant driving force. This result is consistent with the H‐bond interaction between PC and HA reported by Wekwejt et al. [22]. To accurately determine the adsorption capacities of MPNs and excess HA on the PWPI surface at different dosages, the adsorption amounts of MPNs and HA were measured. The mass ratio (m:m) of PWPI to MPNs ranged from 100:5–100:100. As the MPN dosage increased, the amount of MPNs adsorbed on the PWPI surface gradually increased (Figure 2E). However, the adsorption efficiency of MPNs gradually decreased. When the PWPI‐to–MPN ratio was 100:5 or 100:10, almost all the added MPNs were adsorbed onto the PWPI surface (Figure 2F). When the PWPI‐to‐MPN ratio reached ≥ 100:25, the binding efficiency of MPNs decreased significantly (*p* < 0.05). In the reaction system, HA was added in excess. As the amount of MPNs adsorbed on the PWPI surface increased, more HA was adsorbed onto the PWPI@MPNs surface (Figure 2G).

In addition, stability is critical for sustained‐release systems. Therefore, the stability of the SPPE system under different conditions was systematically evaluated. First, differential scanning calorimetry was conducted on PWPI and PWPI@MPNs5/HA–PWPI@MPNs100/HA (Figure 2H). The thermal transition temperature of PWPI was 94.15°C. As the MPN content increased, the thermal transition temperature gradually increased. The PWPI@MPNs100/HA group exhibited the highest thermal transition temperature (111.35°C). This increase may be attributed to two factors. (1) After MPNs were combined with PWPI, the PWPI structure became more ordered and compact (Figure 2C). This structural change stabilized the PWPI conformation and enhanced its resistance to thermal denaturation. In addition, the denser and more compact microstructure restricted the mobility of WP chains. Consequently, protein chain unfolding slowed, resulting in an increased energy barrier. (2) The double‐layer shell composed of polyphenols and polysaccharides protected the encapsulated WP and delayed heat transfer to the core. Compared with WP alone, the sustained‐release system exhibited greater thermal stability. Second, the storage stability of the samples was evaluated using an accelerated stability test. PWPI@MPNs25/HA was prepared in batches and vacuum‐sealed (Figure S10A,B). If the key intermediate MPN coating dissociated into a free state after redispersion in water, the sample was considered to have reached its maximum storage period. Under 65°C and 75% relative humidity, the MPN coating showed significant dissociation after 12 days of storage (*p* < 0.05) compared with day 0 (Figure S11A,B). Therefore, the storage period of the sample at 65°C was determined to be 9 days. At 55°C, the storage period was 24 days (Figure S12A,B). Based on the accelerated stability equation, the storage period at 25°C was estimated to be approximately 455.1 days. The relatively large particle size of PWPI (∼50 µm) may explain the absence of significant changes in particle size during storage (*p* > 0.05) at either 55°C or 65°C (Figures S11C and S12C). Third, the stability of the sample under acidic conditions was evaluated. After PWPI@MPNs25/HA was dispersed in simulated gastric fluid without digestive enzymes, the suspension was incubated on a shaker for 2 h. No significant differences were observed in the contents of free PC or calcium ions before and after incubation (*p* > 0.05; Figure S13A,B), indicating that the sample remained stable under acidic conditions. Fourth, the stability of the SPPE system in the gastrointestinal tract was evaluated. Here, the main purpose was to test whether the SPPE system could withstand the shear force and squeezing force generated by gastrointestinal peristalsis. To eliminate the influence of digestive enzymes, PWPI@MPNs100/HA was used for testing. After oral gavage of PWPI@MPNs100/HA, mice were sacrificed at different time points, and gastrointestinal contents were collected. Within 8 h, more than half of the PC and calcium ions administered via gavage were recovered (Figures S14A and S15A). The proportions of free PC and calcium ions remained relatively low (Figures S14B and S15B), indicating that the sample remained stable during gastrointestinal transit. Overall, these findings indicate that the SPPE system exhibited good stability.

### 2.4 Sustained‐Release Effect of Polymerized Whey Protein Isolate in an In Vitro Digestion Model

**FIGURE 3:**
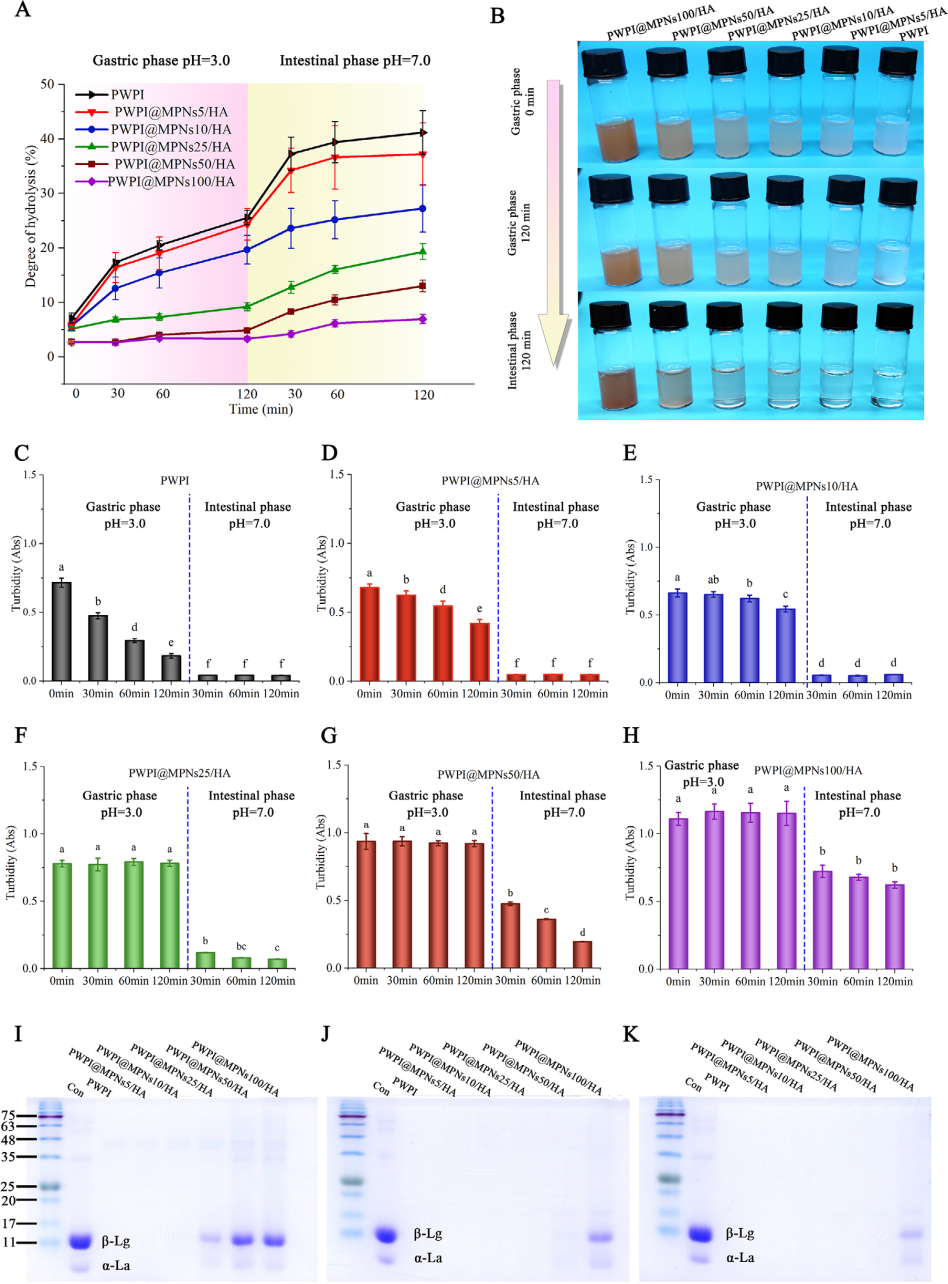
The digestion characterization of PWPI and PWPI@MPNs5/HA‐PWPI@MPNs100/HA during in vitro digestion, including (A) The changes of DH in the gastric and intestinal digestion stages; (B) The digital images of the digestive juices of gastric phase 0 min, gastric phase 120 min, and intestinal phase 120 min; (C–H) The turbidity of the digestive juices of PWPI and PWPI@MPNs5/HA‐PWPI@MPNs100/HA at different times; (I–K) SDS‐page images of digestive juices after gastric digestion for 2 h, intestinal digestion for 2 h, and intestinal digestion for 8 h. Mean ± SE of *n* = 3 (at least) independent experiments. Different lowercase letters indicated significant differences within groups (*p* < 0.05).

To evaluate the effect of different MPNs/HA dosages on the sustained‐release behavior of PWPI, PWPI and PWPI@MPNs5/HA–PWPI@MPNs100/HA were subjected to in vitro digestion, and Figure 3A shows the results. Without the protection of the MPN/HA shell, PWPI was rapidly hydrolyzed. In particular, it was almost completely hydrolyzed after 30 min of intestinal digestion. As the MPNs/HA dosage increased, the release rate of PWPI gradually decreased. In particular, when the PWPI‐to–MPN ratio reached 100:100, PWPI was barely released or digested, indicating that the MPN/HA shell effectively protected PWPI. Digital images showed that at the initial stage of gastric digestion (0 min), PWPI and PWPI@MPNs5/HA–PWPI@MPNs100/HA were opaque (Figure 3B). After 120 min of gastric digestion, PWPI became transparent, while PWPI@MPNs5/HA–PWPI@MPNs100/HA remained opaque. After 120 min of intestinal digestion, PWPI and PWPI@MPNs5/HA–PWPI@MPNs25/HA became completely transparent, while PWPI@MPNs50/HA–PWPI@MPNs100/HA remained turbid. Figure S16 shows the digital images collected at each sampling time point throughout the digestion process. As the MPNs/HA dosage increased, the digestive fluids became more turbid at each sampling time point. To quantitatively assess the degree of opacity, the turbidity of the digestive fluids was measured. The results were consistent with the observations described above (Figure 3C–H). To further evaluate the sustained‐release performance of the system over a longer digestion period, the internal digestion time was extended to 8 h (Figure S17). During this period, the degree of hydrolysis (DH) of PWPI and PWPI@MPNs5/HA showed little change, and the turbidity remained unchanged. This finding may be attributed to the complete hydrolysis of PWPI during the early stage of intestinal digestion. The DH of PWPI in the PWPI@MPNs10/HA–PWPI@MPNs50/HA groups increased to different extents. In the PWPI@MPNs100/HA group, the DH of PWPI remained very low throughout the digestion process, even after 8 h of intestinal digestion. The turbidity results (Figure S18) were consistent with these findings. After 480 min of intestinal digestion, only the PWPI@MPNs50/HA and PWP–I@MPNs100/HA groups retained relatively high turbidity, while the turbidity of the remaining groups was nearly zero. SDS‐PAGE analysis was then conducted after 2 h of gastric digestion, followed by 2 or 8 h of intestinal digestion. After 2 h of gastric digestion, the α‐lactalbumin (α‐La) and β‐lactoglobulin (β‐Lg) bands were barely detectable in PWPI and PWPI@MPNs5/HA–PWPI@MPNs10/HA groups (Figure 3I). Compared with the undigested PWPI (Con) group, the α‐La and β‐Lg bands in the PWPI@MPNs25/HA group were less intense. After 2 and 8 h of internal digestion, the α‐La and β‐Lg bands remained detectable in the PWPI@MPNs100/HA group (Figure 3J,K), indicating that PWPI@MPNs100/HA protected PWPI from proteolytic degradation throughout the digestion process. Gel permeation chromatography was used to compare the molecular weight (MW) distribution after digestion (Table S4). After digestion of PWPI, the MW was mostly below 500 Da (53.89%). The MW of PWPI@MPNs100/HA after digestion below 500 Da was only 37.01%. The above results suggest that MPNs/HA could control the release of PWPI for a long time.

### 2.5 Digestion and Absorption of PWPI@MPNs/HA in the Mouse Gastrointestinal Tract

**FIGURE 4:**
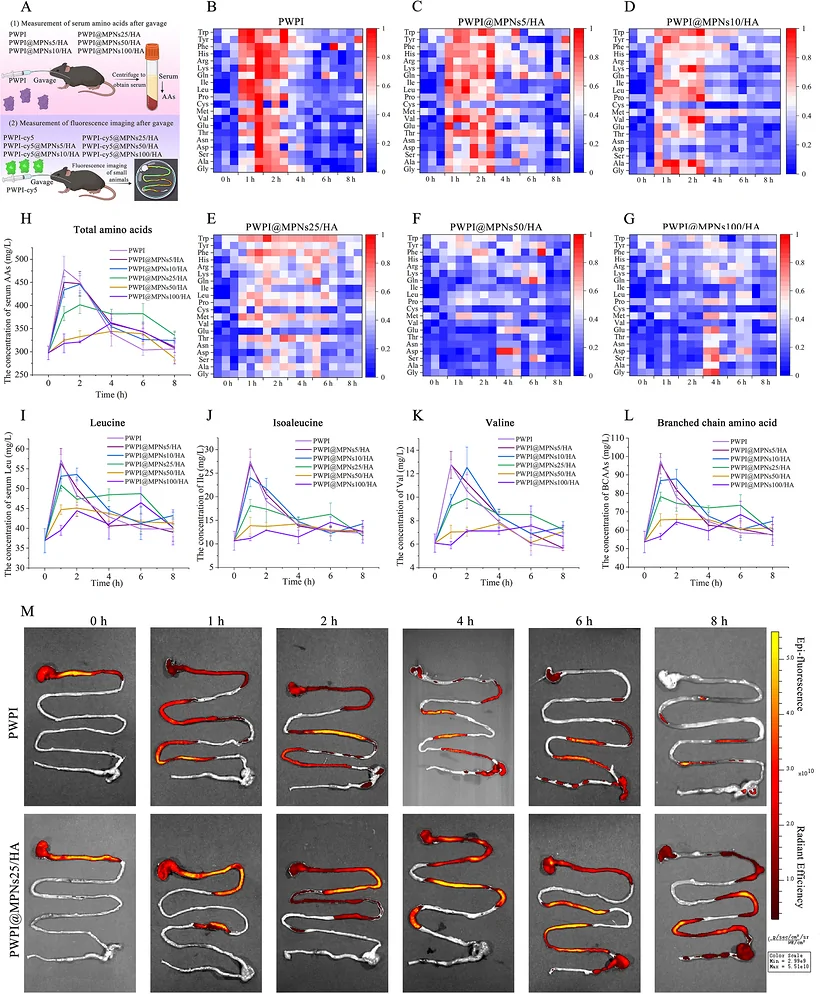
(A) Schematic diagram of serum AAs and fluorescence imaging experiments of small animals. Heatmap of changes in serum AAs after gavage of (B) PWPI, (C) PWPI@MPNs5/HA, (D) PWPI@MPNs10/HA, (E) PWPI@MPNs25/HA, (F) PWPI@MPNs50/HA, and (G) PWPI@MPNs100/HA within 0–8 h; (H–L) The changes of total serum AAs, leucine, isoleucine, valine and BCAAs during the period of 0–8 h after gavage, respectively; (M) After gavage of PWPI‐Cy5 and PWPI‐Cy5@MPNs25/HA, gastrointestinal tracts were taken to observe the digestion of the proteins. Mean ± SE of *n* = 3 (at least) independent experiments.

Next, serum AAs levels and fluorescence imaging were evaluated after oral gavage (Figure 4A). After oral gavage of PWPI, serum AA levels peaked within 1–2 h (Figure 4B), indicating rapid digestion of PWPI, consistent with previous studies [23]. The serum AAs peaks in the PWPI@MPNs5/HA and PWPI@MPNs10/HA groups were reduced at 1–2 h and increased at 4–6 h (Figure 4C,D). After oral gavage of PWPI@MPNs25/HA, the peak serum AA level decreased significantly at 1–2 h and increased significantly at 4–6 h (Figure 4E). In the PWPI@MPNs50/HA (Figure 4F) and PWPI@MPNs100/HA (Figure 4G) groups, serum AA levels remained low throughout the digestion period. Figure 4H shows the total serum AA concentrations. The results showed that PWPI@MPNs25/HA sustained protein release during the digestion process, maintaining high serum AA levels for 1–6 h after oral gavage. This duration was 400% longer than the 1–2 h serum AA peak observed for PWPI. The BCAAs in WP include leucine, isoleucine, and valine, particularly leucine, which plays a critical role in promoting MPS [24, 25]. PWPI@MPNs25/HA prolonged the elevation of serum concentrations of these three BCAAs and their combined concentration (Figure 4I–L). Additionally, to demonstrate the advantages of the SPPE system, it was compared with casein (CAS) and cross‐linked WP (CWPI). CWPI was prepared using TGase at 50°C for 2 h. The particle size of CWPI was 722.7 nm (Figure S19A), significantly larger than that of WPI (*p* < 0.01; Figure S19B). After oral gavage of an equivalent dose of CAS or CWPI to that of PWPI@MPNs25/HA (0.84 g/kg), serum AA levels in both groups increased at 1–2 h and decreased significantly at 3 h (Figure S20A,B). The total serum AA concentration also decreased significantly at 3 h (Figure S20C). PWPI@MPNs25/HA extended the sustained‐release duration of these conventional formulations by at least 100%. To further correlate the sustained release of WP with changes in serum AA levels, the same dose of WP was divided into six equal portions and administered via oral gavage at 1 h intervals. This treatment group was designated EWPI. Figure S21A,B shows that the serum AA profile of the EWPI group closely resembles that of the PWPI@MPNs25/HA group.

To evaluate the gastrointestinal digestion of PWPI@MPNs/HA, PWPI@MPNs25/HA was selected as the representative formulation for fluorescence imaging (Figure 4M). After oral gavage of PWPI, strong fluorescence signals were observed at 1 and 2 h. By 4 h, the fluorescence intensity had decreased significantly, suggesting that PWPI was almost completely digested within 4 h. In contrast, PWPI@MPNs25/HA still exhibited a strong fluorescence signal at 6 h. Although the fluorescence intensity decreased at 8 h, it remained significantly higher than that of PWPI. The statistical analysis of fluorescence intensity also reflects this phenomenon (Figure S22). In addition, PWPI reached the cecum at 4 h, whereas PWPI@MPNs25/HA reached the cecum at 6 h, representing an approximately 2–h delay. These findings further indicate that the MPN/HA shell prolonged the gastrointestinal retention of PWPI. This effect may be attributed to the strong gastrointestinal mucoadhesive properties of MPNs and HA [26, 27], increasing the residence time of WPI in the gastrointestinal tract.

### 2.6 Evaluation of the Therapeutic Effect of PWPI@MPNs/HA in a Murine Sarcopenia Model

**FIGURE 5:**
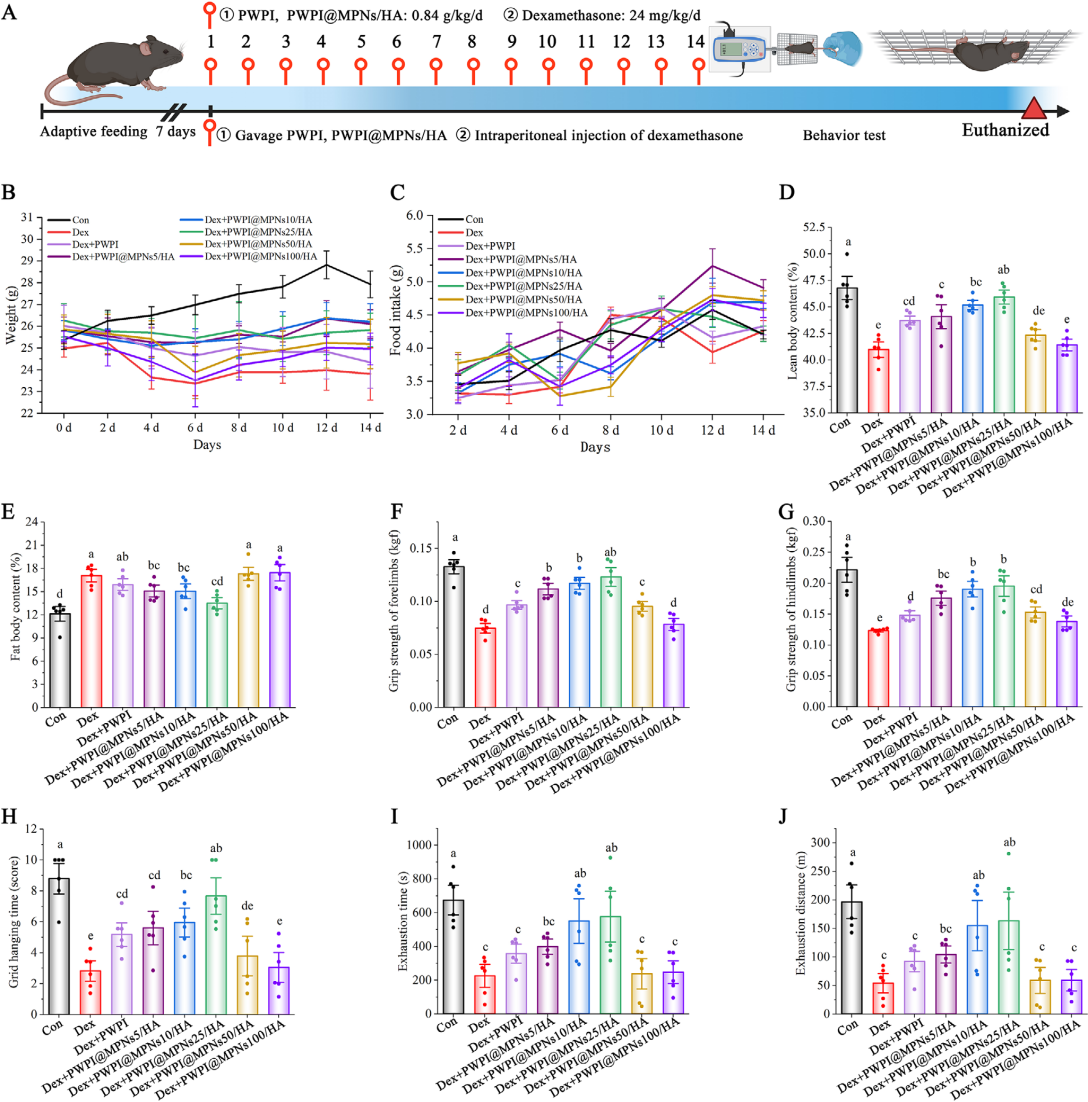
(A) Scheme design for sarcopenia model establishment and PWPI@MPNs/HA treatment; (B) Weight changes during the sarcopenia model establishment period; (C) Food intake changes in the sarcopenia model establishment period; (D) lean body content, (E) fat body content, (F) forelimb grip strength, (G) hindlimb grip strength, (H) grid hanging time score, (I) exhaustion time, and (J) exhaustion distance of the mice were measured after 14 days of intraperitoneal Dex injection and treatment. Mean ± SE of *n* = 6 (at least) independent experiments. Different lowercase letters indicated significant differences within groups (*p* < 0.05).

To evaluate the therapeutic effect of PWPI@MPNs/HA, a dexamethasone (Dex)‐induced sarcopenia model was established (Figure 5A). Sarcopenia induction and treatment were initiated simultaneously and continued for 14 days. During this period, the body weight of the Con group gradually increased (Figure 5B). However, compared to the Con group, all groups that received intraperitoneal Dex injections lost weight, with the Dex group showing the greatest reduction. Compared to the Dex group, the other Dex‐treated groups showed slight recovery. Dex‐induced skeletal muscle atrophy via the combined activation of the ubiquitin–proteasome system (UPS) and inhibition of the mammalian target of rapamycin (mTOR) pathway, ultimately leading to weight loss [28]. During the intervention period, food intake increased over time, with no significant differences among the groups (e.g., Con vs. Dex) (Figure 5C). Similarly, Wang et al. report that mice with Dex‐induced sarcopenia were fed goat WP, goat casein, bovine WP, and bovine casein. No significant differences in food intake were observed during the 8‐week intervention period [29]. After the 14‐day intervention, the lean body and fat body contents were measured (Figure 5D,E). Compared to the Con group, the Dex group showed a significant decrease in lean body content and a significant increase in fat body content (*p* > 0.05). Lean body and fat body masses showed the same trend (Figure S23). Treatment with PWPI and PWPI@MPNs5/HA‐PWPI@MPNs25/HA effectively reversed these changes. The PWPI@MPNs50/HA‐PWPI@MPNs100/HA group showed no significant differences in lean body or fat body contents compared to the Dex group (*p* > 0.05). This finding suggests that the higher MPNs/HA shell content may have slowed PWPI release, thereby reducing its therapeutic effect. In subsequent behavioral tests, including forelimb grip strength (Figure 5F), hindlimb grip strength (Figure 5G), grid hanging time (Figure 5H), exhaustion time (Figure 5I), and exhaustion distance (Figure 5J), the Dex group exhibited significantly impaired muscle function. These findings confirm the successful establishment of the sarcopenia model. As the MPNs/HA dosage increased, muscle function initially improved and then declined. The PWPI@MPNs25/HA group showed the best performance, with significantly better muscle performance than the Dex group.

**FIGURE 6:**
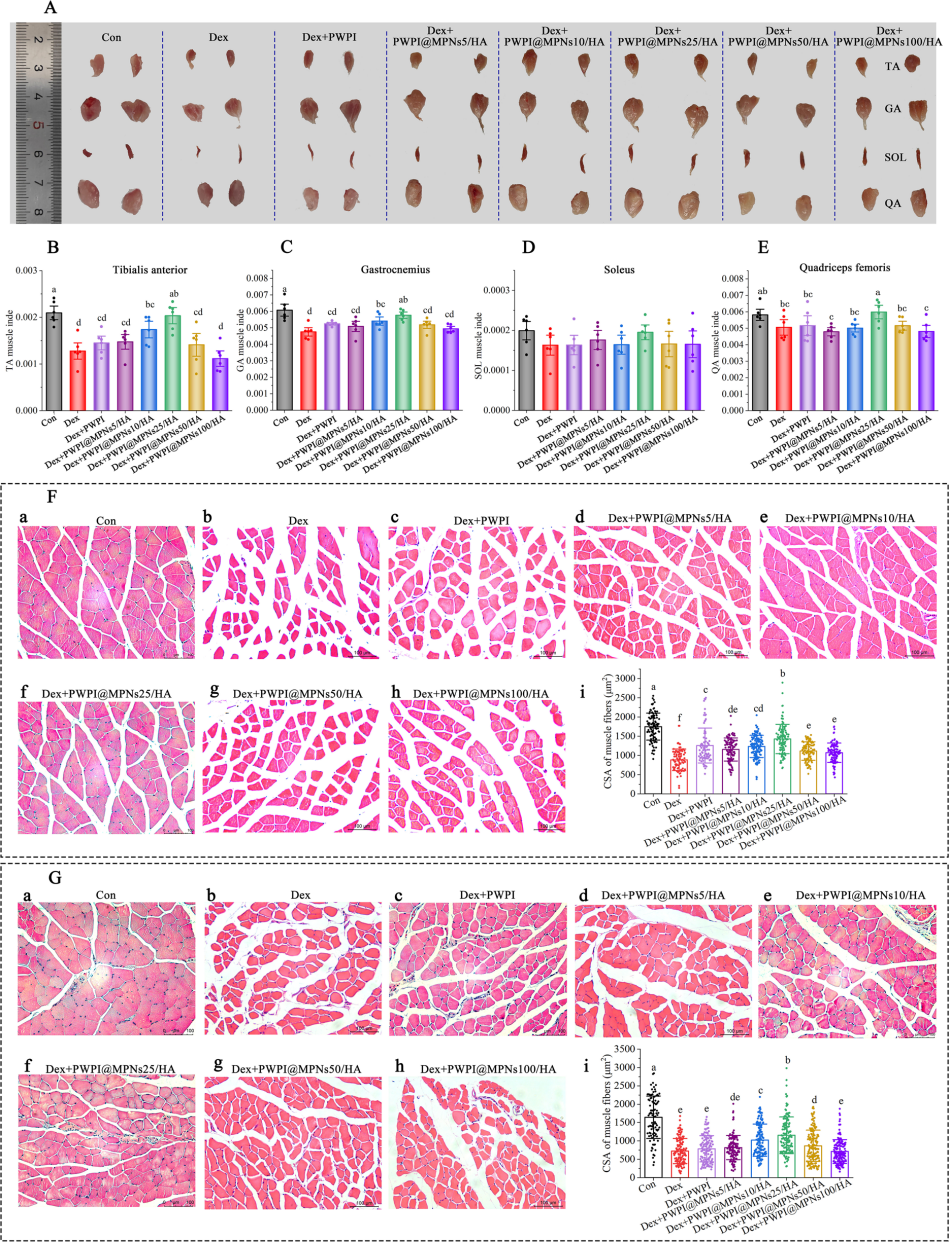
(A) The digital images of TA, GA, SOL and QA of the hindlimbs; The muscle index of TA (B), GA (C), SOL (D), and QA (E); (F) HE staining images and the CSA statistical analysis results of GA; (G) HE staining images and the CSA statistical analysis results of TA. Mean ± SE of *n* = 6 (at least) independent experiments. Different lowercase letters indicated significant differences within groups (*p* < 0.05).

After euthanasia, the tibialis anterior (TA), gastrocnemius (GA), soleus (SOL), and quadriceps femoris (QA) muscles were collected for gross examination and measurement. Figure 6A illustrates that Dex treatment caused marked atrophy in all four types of muscles. Treatment with PWPI and PWPI@MPNs5/HA‐PWPI@MPNs25/HA progressively alleviated muscle atrophy and increased muscle size. In the PWPI@MPNs50/HA‐PWPI@MPNs100/HA group, muscle atrophy remained significant. Subsequently, the muscles were weighed, and the skeletal muscle index was calculated by normalizing muscle weight to body weight, providing a more accurate assessment of these changes (Figure 6B–E). However, the SOL showed no significant differences among the groups. This may be attributed to the small mass of the SOL, potentially increasing measurement variability and affecting the experimental results. The GA and TA are widely regarded as representative skeletal muscles for evaluating muscle strength and functional status, particularly the GA [30, 31]. Therefore, the GA and TA tissues were stained with hematoxylin and eosin (H&E), and muscle fiber cross‐sectional area (CSA) was evaluated and quantified. The H&E staining of the GA showed that muscle fibers in the Con group had a larger CSA and a well‐organized architecture (Figure 6Fa). In the Dex group, muscle fiber CSA was significantly reduced, and the fibers exhibited a disorganized arrangement (Figure 6Fb). Treatment with PWPI and PWPI@MPNs5/HA alleviated muscle fiber atrophy (Figure 6Fc,d). The PWPI@MPNs10/HA‐PWPI@MPNs25/HA group showed greater inhibition of muscle fiber atrophy (Figure 6Fe,f). Quantitative analysis of muscle fiber CSA revealed that the PWPI@MPNs25/HA group exhibited the highest recovery among all Dex‐treated groups (Figure 6Fi). However, the PWPI@MPNs50/HA‐PWPI@MPNs100 group showed significant muscle fiber atrophy and disorganized fiber architecture (Figure 6Fg,h). These findings suggest that these treatments provide limited protection against sarcopenia. Figure 6Ga–i shows the H&E staining images and quantitative analysis of the TA. The TA showed a histological pattern similar to that of the GA. In the PWPI and PWPI@MPNs5/HA‐PWPI@MPNs10/HA groups, WP was released rapidly, with serum AA concentrations peaking approximately 1 h after gavage. This rapid release may have limited the increase in muscle fiber CSA. In contrast, the PWPI@MPNs25/HA group provided stable, sustained WP release, maintaining serum AA concentrations for approximately 6 h. Even at 8 h, serum AA concentrations remained higher than those in all other gavage groups. This prolonged AA stimulation inhibited muscle fiber atrophy. In the PWPI@MPNs50/HA‐PWPI@MPNs100/HA group, serum AA concentrations showed minimal fluctuations throughout the postprandial period. Consequently, muscle fiber CSA continued to decrease and remained significantly lower than that of the Con group. Overall, maintaining effective serum AA concentrations over an extended period may help preserve muscle fiber CSA.

### 2.7 Molecular Mechanisms Underlying the Observed Effects

**FIGURE 7:**
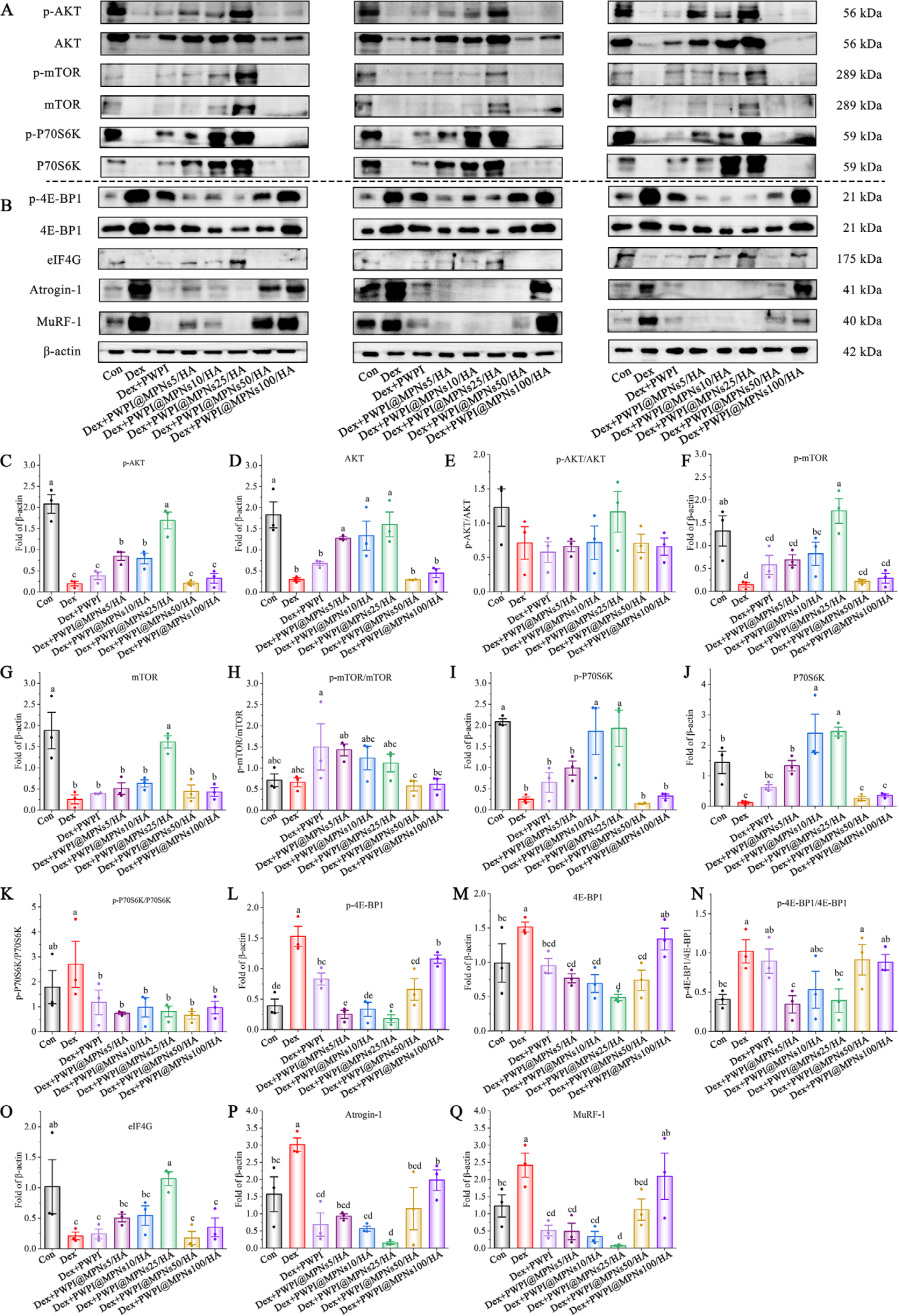
(A) Results of WB on molecular pathways of MPS; (B) Results of WB on molecular pathways of MPB; The results of semi‐quantitative analysis of WB bands include (C) p‐AKT, (D) AKT, (E) p‐AKT/AKT, (F) p‐mTOR, (G) mTOR, (H) p‐mTOR/mTOR, (I) p‐P70S6K, (J) P70S6K (K) p‐P70S6K/P70S6K, (L) p‐4E‐BP1, (M) 4E‐BP1, (N) P‐4E‐BP1/4E‐BP1, (O) eIF4G, (P) *Atrogin‐1*, and (Q) *MuRF‐1*. Mean ± SE of *n* = 3 (at least) independent experiments. Different lowercase letters indicated significant differences within groups (*p* < 0.05).

**FIGURE 8:**
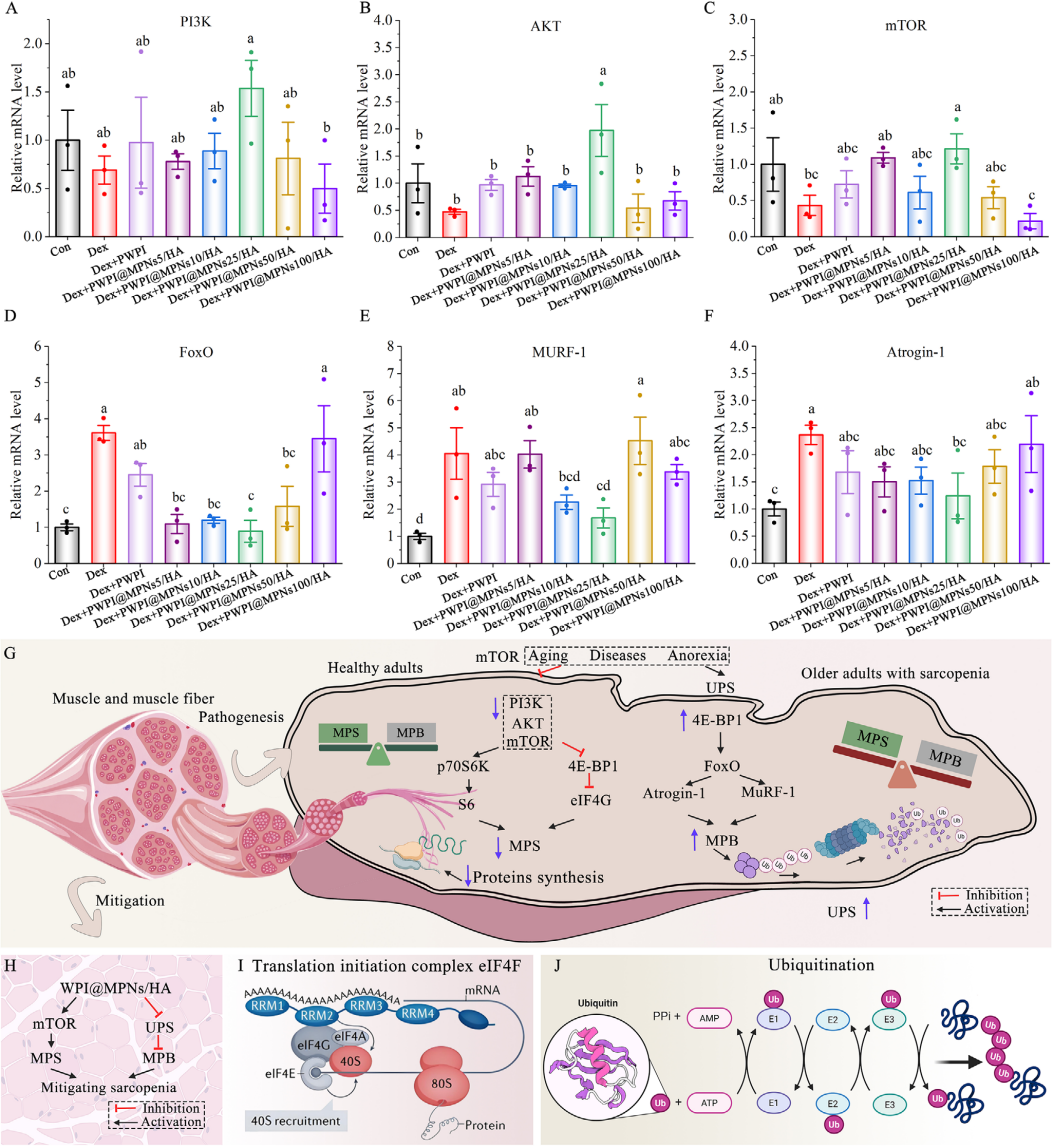
The results of quantification of key genes include (A) *PI3K*, (B) *AKT*, (C) *mTOR*, (D) *FoxO*, (E) *MuRF‐1*, and (F) *Atrogin‐1*; (G) Schematic diagram of the pathogenesis of sarcopenia. In the healthy human body, the rates of MPB and MPS are in a dynamic equilibrium state. During the development of sarcopenia, the MPS pathway of PI3K/AKT/mTOR is inhibited, while the MPB pathway (UPS) is enhanced. This leads to the rate of MPB being greater than the rate of MPS, ultimately causing sarcopenia; (H) PWPI@MPNs/HA can enhance the PI3K/AKT/mTOR signaling pathway and weaken the UPS signaling pathway. It once again brings the rate of MPB and MPS into a dynamic equilibrium state. Ultimately, it resisted the occurrence and development of sarcopenia. (I) Schematic diagram of the relationship among eIF4G, eIF4A and eIF4E; (J) Schematic diagram of the ubiquitination process. Mean ± SE of *n* = 3 (at least) independent experiments. Different lowercase letters indicated significant differences within groups (*p* < 0.05).

Muscle protein anabolism and catabolism are crucial for the onset and progression of sarcopenia. Sustained MPS rates lower than MPB rates promote the development of sarcopenia [5]. Among the pathways regulating protein anabolism, the Phosphoinositide 3‐kinase (PI3K)/protein kinase B (AKT)/mTOR signaling pathway is a key regulator. PI3K phosphorylates phosphatidylinositol 4,5‐bisphosphate at the plasma membrane to generate phosphatidylinositol 3,4,5‐trisphosphate (PIP3) [32]. As a second messenger, PIP3 binds to AKT and promotes signaling that stimulates MPS [33]. AKT is a major downstream effector of PI3K. It is a cytoplasmic serine/threonine kinase that contains a pleckstrin homology (PH) domain, a catalytic domain, and a regulatory domain [34]. The Con group exhibited high p‐AKT expression, while the Dex group showed significantly reduced p‐AKT expression (Figure 7A). Among the PWPI and PWPI@MPNs5/HA‐PWPI@MPNs25/HA groups, p‐AKT expression increased with increasing MPNs/HA content. In contrast, the PWPI@MPNs50/HA‐PWPI@MPNs100/HA group exhibited significantly reduced p‐AKT expression. Semiquantitative densitometric analysis of the immunoblot bands revealed that the PWPI@MPNs25/HA group exhibited the highest p‐AKT expression among all Dex‐treated groups (Figure 7C). Additionally, total AKT expression and the p‐AKT/AKT ratio exhibited the same pattern (Figure 7D,E). Upon activation, AKT translocates to the plasma membrane and phosphorylates multiple downstream targets, including mTOR and Forkhead box O (FoxO) proteins [35]. mTOR is a central regulator of protein synthesis, promoting translocation by phosphorylating downstream targets, including 70 kDa ribosomal protein S6 kinase (P70S6K) and eukaryotic translation initiation factor 4E‐binding protein 1 (4E‐BP1) [36]. Treatment with PWPI and PWPI@MPNs5/HA‐PWPI@MPNs25/HA significantly increased p‐mTOR expression (Figure 7F). The PWPI@MPNs25/HA group also exhibited significantly higher total mTOR expression and p‐mTOR/mTOR ratio than the Dex group (Figure 7G,H). Furthermore, studies show that AAs activate mTORC1 via Rag guanosine triphosphatases [37]. Leucine, a major AA in WP, binds to Sestrin2, reduces its interaction with GATOR2, and ultimately activates mTORC1 [38]. This mechanism is consistent with the increased mTOR expression observed in this study. p70S6K is a key downstream effector of protein synthesis. It enhances mRNA translation by phosphorylating ribosomal protein S6, thereby increasing the protein synthesis capacity of the cell [39]. In the PWPI@MPNs10/HA‐PWPI@MPNs25/HA group, p‐P70S6K expression, total P70S6K expression, and the p‐P70S6K/P70S6K ratio increased significantly (Figure 7I–K), indicating that sustained PWPI release at an appropriate rate could reverse the Dex‐induced decrease in MPS. Protein synthesis involves four stages: initiation, elongation, termination, and ribosome recycling [40]. Among these, translation initiation is the primary rate‐limiting step. Eukaryotic translation initiation factor* 4* gamma (eIF4G) is a large scaffold protein that serves as a docking platform during eukaryotic mRNA translation initiation and mediates cap‐dependent and cap‐independent translation initiation [41]. eIF4G recruits the cap‐binding protein eIF4E and the RNA helicase eIF4A to form the eIF4F translation initiation complex (Figure 8I) [42]. Compared to the Con group, eIF4G expression was significantly reduced in the Dex group, indicating suppression of translation initiation (Figure 7B). Treatment with PWPI@MPNs25/HA significantly increased eIF4G expression compared to the Dex group (*p* < 0.05) (Figure 7O), suggesting restoration of translation initiation. A translational repressor that binds tightly to eIF4E in its unphosphorylated state (4E‐BP1), competitively preventing its interaction with eIF4G [43]. In the PWPI@MPNs25/HA group, p‐4E‐BP1 expression, total 4E‐BP1 expression, and the p‐4E‐BP1/4E‐BP1 ratio were all reduced (Figure 7L–N), indicating reduced inhibition of eIF4G and restoration of translation initiation. These findings indicate that sustained WP release prolongs effective serum AA concentrations and continuously activates the PI3K/AKT/mTOR/p70S6K signaling pathway. This response was greater than that of transient stimulation with higher serum AA concentrations.

UPS is a major pathway for protein degradation. Ubiquitination involves three sequential enzymatic reaction steps: E1 activates ubiquitin and transfers it to E2, E2 transfers ubiquitin to E3, and E3 catalyzes the covalent attachment of ubiquitin to lysine residues on the target protein (Figure 8J) [44]. Ubiquitin–protein ligase E3 binds directly or indirectly to substrate proteins and facilitates the transfer of ubiquitin from the E2–ubiquitin thioester intermediate to the ε‐amino group of lysine residues on target proteins, forming an isopeptide bond [45]. RING finger 1 (MuRF‐1) and muscle atrophy F‐box protein (Atrogin‐1/FBXO32) are muscle‐specific E3 ubiquitin ligases crucial for the regulation of muscle atrophy [46]. The Dex group exhibited significantly increased expression of the muscle atrophy markers, *MuRF‐1* and *Atrogin‐1* (Figure 7B), indicating enhanced muscle protein degradation. Treatment with PWPI and PWPI@MPNs5/HA‐PWPI@MPNs25/HA significantly reduced *MuRF‐1* and *Atrogin‐1* expression (Figure 7P,Q). No significant differences were observed between the PWPI@MPNs50/HA‐PWPI@MPNs100/HA group and the Dex group in the MPS or MPB signaling pathways, suggesting that an excessively thick shell limits PWPI release and consequently reduces its ability to alleviate sarcopenia. Overall, the SPPE system maintained effective serum AA concentrations over an extended period, resulting in continuous inhibition of the UPS, a key mechanism underlying its therapeutic effect.

To further confirm the changes in the MPS and MPB signaling pathways, the expression of the key genes was quantified. The expression of genes in the *PI3K/AKT/mTOR* signaling pathway was significantly reduced in the Dex group compared to that in the Con group (Figure 8A–C). In the PWPI@MPNs25/HA group, the expression of these genes increased significantly, indicating that the SPPE system enhances the MPS signaling and attenuates muscle atrophy. *AKT* phosphorylates *FoxO*, reducing its DNA‐binding affinity while increasing its interaction with cytoplasmic binding proteins [47]. Consequently, *FoxO* translocates from the nucleus to the cytoplasm, reducing its transcriptional activity and the expression of *FoxO* target genes [48]. Ultimately, the expression of muscle‐specific E3 ubiquitin ligases, such as *MuRF‐1* and *Atrogin‐1*, is reduced. *FoxO* gene expression was significantly higher in the Dex group than in the Con group (Figure 8D; *p* < 0.05), promoting the expression of downstream muscle‐specific ubiquitin ligases and ultimately increasing MPB. As the MPNs/HA dosage increased, *FoxO* gene expression initially decreased and then increased, indicating that an appropriate PWPI release rate more effectively suppressed MPB. *MuRF‐1* and *Atrogin‐1* expression was significantly reduced in the PWPI@MPNs25/HA group, indicating suppression of the MPB (Figure 8E,F). Overall, the quantitative gene expression results were consistent with the corresponding protein expression measured using Western blot analysis.

### 2.8 Preparation of Polymerized Whey Protein Isolate

The WPI used in this study was Hilmar 9410 (WPI Hilmar 9410), with a particle size of 231.23 nm (Figure S24). Polymerizing WPI into larger PWPI may reduce its specific surface area, thereby reducing the amount of the MPNs/HA shell required. During polymerization, the WPI was heated at pH 7 and 90°C for different durations (0–120 min). After 20 min of heating, the particle diameter increased to approximately 300 nm and remained stable. Extending the heating time to 120 min increased the particle size to 312 nm; however, this increase was not significantly different from that observed after 20 min (*p* < 0.05; Figure S25). Therefore, heat treatment alone was insufficient to polymerize WPI into micrometer‐sized or larger particles. The results showed that adjusting the pH to near the pI of WPI before heating significantly increased the particle size (Figure S2). Therefore, acid‐assisted heat treatment was essential for polymerizing WPI into micrometer‐sized particles. This may be attributed to the minimal electrostatic repulsion between WPI molecules near the pI. Heat‐induced unfolding exposed the hydrophobic groups, promoting WPI aggregation, hydrophobic interactions, and disulfide bond formation to generate polymerized PWPI particles (Figures S6–S8).

### 2.9 Mechanism of Protein Encapsulation and Release in the Single‐Protein Particle Encapsulation System

MPNs are supramolecular structures formed through the coordination of metal ions with polyphenols containing *ortho*‐dihydroxyl groups (Figure S26A), 5‐hydroxyl and/or 3‐hydroxyl groups, and C4 ketone groups (Figure S26B,C) [49]. Under neutral conditions, MPNs remain relatively stable. However, under acidic conditions, partial protonation of the phenolic hydroxyl groups in MPNs destabilizes the metal–phenolic coordination, resulting in network disassembly [17]. Therefore, an additional HA layer was self‐assembled onto the MPN surface to protect the encapsulated PWPI particle from gastric acid and proteolytic digestion. Increasing the MPN dosage promoted greater HA adsorption onto the MPN surface (Figure 2E–G). In vitro digestion experiments confirmed the controlled release of PWPI (Figure 3A). Therefore, the proposed mechanism likely involved different MPN dosages exposing different surface areas of individual PWPI particles to proteases (Figure S27A). Higher MPNs/HA dosages reduced the exposed surface area, limiting protease access to the encapsulated PWPI and slowing its digestion (Figure S27B). At a PWPI‐to‐MPN ratio of 100:100, the external MPNs/HA barrier effectively prevented the proteases from digesting the encapsulated PWPI particles.

### 2.10 Role of the MPNs/HA Shell

In this study, PC, Ca^2+^, and HA were selected because these components mitigate sarcopenia [50, 51, 52, 53]. Additionally, PC and Ca^2+^ are recommended for dietary intake in the “Dietary Reference Intakes for Chinese Residents (2023 Edition).” HA has been approved by the National Health Commission of China as a novel food ingredient for use in milk, dairy products, beverages, and other food products. In the PWPI@MPNs25/HA sample, PWPI, MPNs, and HA accounted for 75.97%, 14.30%, and 9.73% (w/w), respectively. Therefore, beyond providing sustained PWPI release, MPNs/HA may also contribute to alleviating sarcopenia. To evaluate this possibility, a Dex+MPNs/HA group was included. This group differed from the Dex+PWPI@MPNs25/HA group only in the absence of PWPI. Figure S28A presents the treatment design. The Dex+MPNs/HA group showed no significant difference in body weight compared to the Dex group, but had significantly lower body weight than the Con group (Figure S28B). Similarly, food intake did not differ significantly among the MPNs/HA, Con, and Dex groups (Figure S28C). The MPNs/HA group showed no significant differences from the Dex group in lean body content, fat body content, lean body mass, fat body mass, forelimb grip strength, hindlimb grip strength, grid hanging time, exhaustion time, or exhaustion distance (Figure S28D–L). Furthermore, treatment with MPNs/HA did not restore the size or muscle index of the TA, GA, SOL, or QA muscles (Figure S29A–E). H&E staining of the GA and TA muscles showed that the CSA of muscle fibers in the MPNs/HA group was smaller than that in the Con group and did not differ significantly from that in the Dex+MPNs/HA group (*p* > 0.05) (Figure S29F,G). These findings indicate that MPNs/HA alone have no therapeutic effect on sarcopenia, potentially due to MPNs/HA aggregation, which may have limited their biological activity. This mechanism warrants further investigation in future studies. Encapsulation with MPNs/HA significantly prolonged the gastrointestinal retention time of PWPI@MPNs/HA (Figure 4M). For example, PWPI reached the cecum at 4 h and PWPI@MPNs25/HA at 6 h, representing a delay of approximately 2 h. Therefore, MPNs/HA may serve two functions: (1) controlling the release and digestion rate of PWPI and (2) prolonging its gastrointestinal retention time. Overall, MPNs/HA may enhance the therapeutic efficacy of PWPI against sarcopenia.

### 2.11 Molecular Mechanism in Sarcopenia

There are many factors that can trigger sarcopenia, such as aging, anorexia nervosa, long‐term bed rest due to illness, or lack of exercise [1]. In the occurrence and development of sarcopenia, the MPB and MPS play a crucial role. When the rate of MPB is greater than MPS, it will cause muscle loss (Figure 8G). Among the anabolic pathways of proteins, PI3K/AKT/mTOR is a key metabolic pathway. An important pathway for MPB is the UPS. The results of WB and PCR showed that Dex could inhibit PI3K/AKT/mTOR and enhance the UPS signaling pathway. This eventually led to the occurrence of sarcopenia. PWPI@MPNs25/HA could inhibit the action of Dex, enhance the PI3K/AKT/mTOR signaling pathway, and suppress the UPS signaling pathway, achieving a balance between protein synthesis and decomposition, and ultimately resisting the occurrence and development of sarcopenia (Figure 8H).

### 2.12 Potential Applications of the Single‐Protein Particle Encapsulation System

In the SPPE system, MPNs serve as an intermediate bridging layer to encapsulate a single PWPI particle and anchor HA to the outer surface. In this study, the self‐assembly mechanism between MPNs and PWPI was investigated, showing that H‐bonds and hydrophobic interactions are the primary intermolecular forces. Studies report that polyphenols bind to proteins via multipoint hydrogen bonding [49]. Polyphenols bind to peptide groups in the protein backbone via bidentate H‐bonds formed by their *ortho*‐phenolic hydroxyl groups. One phenolic hydroxyl group served as the H donor, while the carbonyl oxygen of the peptide group served as the H acceptor (Figure S26D). Additionally, gallic acid moieties are hydrophobic and can form hydrophobic associations with hydrophobic regions of protein molecules. Leucine, valine, alanine, proline, and other amino acid residues contribute to the hydrophobic domains of proteins, promoting polyphenol binding to protein peptide backbones. Therefore, the SPPE system developed in this study may protect different proteins, regardless of their hydrophilicity, hydrophobicity, PI, or spatial structure. In the future, this system may be used to protect various proteins, such as lactoferrin and immunoglobulins, and facilitate their delivery to the intestinal tract in an intact form, suggesting broad application potential.

MPNs can be assembled from various polyphenols and metal ions. Commonly used polyphenols for constructing MPNs include epigallocatechin gallate, TA, GA, and polydopamine. Common metal ions include Fe^3+^, Cu^2+^, Ca^2+^, Zn^2+^, and Mg^2+^ [54]. Polyphenols exhibit antioxidant, antibacterial, free radical‐scavenging, immunomodulatory, and neuroprotective activities [54]. These metal ions have significant biological functions, including promoting bone regeneration, exerting antibacterial effects, participating in the synthesis of transport proteins, and serving as catalytic cofactors for essential enzymes [55]. Therefore, MPNs with different compositions can be tailored for treating specific age‐related diseases. Additionally, this study showed that MPNs and HA may interact via H‐bonding. Studies report the self‐assembly of MPNs with various polysaccharides, such as β‐glucan and low‐methoxy pectin [56]. Polysaccharides also exhibit various health‐promoting biological activities. For example, β‐glucan is a common prebiotic widely found in food and plants. Intestinal microbiota metabolize β‐glucan into short‐chain fatty acids with diverse physiological activities [57]. Therefore, the availability and tunability of polyphenols, metal ions, and polysaccharides may facilitate their future use in precision medicine.

### 2.13 Advantages and Limitations

This study shows that the SPPE system has several key advantages: (1) precise control of protein release in the gastrointestinal tract; (2) mild reaction conditions, with the self‐assembly process driven by changes in entropy and enthalpy; (3) safe raw materials that require no chemical cross‐linking reagents or enzymes, thereby avoiding by‐product formation; (4) low‐cost materials and a simple preparation process. To our knowledge, this is the first study to achieve precise control of PWPI release for enhanced mitigation of sarcopenia. The SPPE system developed in this study may have broad potential for future protein protection and delivery applications.

However, some aspects require further investigation. The physiological conditions of older adults with sarcopenia are more complex than those represented by mouse models. For example, older adults with sarcopenia exhibit greater variability in intestinal digestive capacity, anabolic resistance, gastrointestinal transit times, and intestinal pressure. PWPI@MPNs100/HA protected WP from digestion during the 2‐h gastric digestion phase and the 8‐h intestinal digestion phase (Figure S17). Given that human digestion generally occurs over a longer period than in mice, increasing the thickness of the MPNs/HA shell may further extend the sustained‐release period. In the future, administering the SPPE system twice daily, once in the morning and once in the evening, may achieve sustained release for >12 h and maximize its potential to mitigate sarcopenia. Besides, more in‐depth tests should be conducted regarding the stability of the SPPE system, including the accurate storage period at room temperature of 25°C, freezing stability, stability during vehicle transportation, and stability during shelf sales. Additionally, patients with sarcopenia often have comorbidities, such as diabetes, osteoporosis, and chronic organ diseases, which may affect the therapeutic efficacy of PWPI@MPNs/HA. During large‐scale production, WP polymerization and MPNs/HA shell coating may alter the sustained‐release performance due to scale‐up effects. Furthermore, given that no similar products have been developed, the production line requires careful design and optimization. For example, industrial processes, such as stirring, centrifugation, precipitate washing, and drying, may affect the preparation efficiency of the SPPE system. However, as a proof‐of‐concept experiment, the main purpose of this study is to verify the feasibility of the SPPE system. Further comprehensive studies are needed to translate PWPI@MPNs/HA from laboratory proof of concept to clinical application.

## 3 Conclusion

In this study, a novel SPPE system was successfully developed to protect PWPI and precisely control its digestion rate in the gastrointestinal tract, thereby effectively alleviating sarcopenia. In an in vitro digestion experiment, the digestion rate of PWPI was controlled by adjusting the dosage of the intermediate MPN layer. Serum AA measurements after gavage showed that PWPI@MPNs25/HA reduced the peak serum AA concentrations of PWPI and extended the effective AA availability by 400%. To our knowledge, this finding represents the longest sustained‐release period reported for WPI. Additionally, MPNs/HA prolonged the gastrointestinal retention time of PWPI. In animal experiments, increasing MPN dosage initially improved muscle performance in mice with sarcopenia, followed by a decline at higher dosages. These findings indicate that an optimal PWPI release rate produces the greatest therapeutic effect. The molecular mechanisms underlying the effects of PWPI@MPNs/HA were then examined. PWPI@MPNs/HA significantly inhibited the expression of *Atrogin‐1* and *MuRF‐1* in the UPS and enhanced the PI3K/AKT/mTOR signaling pathway, thereby restoring the balance between MPB and MPS and attenuating Dex‐induced muscle atrophy. The SPPE system developed in this study may be broadly applicable to diverse physicochemical properties, making it a potentially universal strategy for protein protection with broad application potential. Furthermore, the interchangeable components of polyphenols, metal ions, and saccharides may enable the development of customized therapies for different diseases.

## 4 Materials and Methods

### 4.1 Materials

WPI9410 (91% protein content) was purchased from Hilmar Corporation (California, USA). HA was purchased from Sinopharm Group Chemical Reagent Co., Ltd. (Shanghai, China). PC, anhydrous calcium chloride, D‐glucuronic acid, carbazole, Foline‐phenol, anhydrous sodium carbonate, dimethyl sulfoxide, magnesium chloride hexahydrate, ammonium carbonate, calcium chloride dihydrate, L‐phenylalanine, and trypsin from porcine pancreas were purchased from the Shanghai Macklin Biochemical Technology Co., Ltd. (Shanghai, China). Rhodamine B, fluorescein isothiocyanate (FITC), α‐amylase, and hematoxylin‐eosin (HE) staining kit were purchased from the Beijing Solaibao Technology Co., Ltd. (Beijing, China). Pepsase, anhydrous borax, *o*‐phthalaldehyde (OPA), and dexamethasone sodium phosphate were purchased from Shanghai Aladdin Biochemical Technology Co., Ltd. (Shanghai, China). Glacial acetic acid, absolute ethyl alcohol, and xylene were purchased from Tianjin Zhiyuan Chemical Reagent Co., Ltd. (Tianjin, China). Methyl alcohol, acetonitrile, and formic acid were purchased from Thermo Fisher Scientific (Massachusetts, USA). Diethyl pyrocarbonate (DEPC) water, 4% tissue cell fixative, sodium dodecyl sulfate (SDS), and phosphate buffer saline (PBS) were purchased from Beijing Feimo Biotechnology Co., Ltd. (Beijing, China). CY5‐SE triethylamine salt was purchased from MedChemExpress (New Jersey, USA). Hypersensitive ECL luminescent solution and Minute Block Rapid Low Background Blocking Fluid were purchased from Affinibody LifeScience (Wuhan, China). TB Green Premix Ex Tag was purchased from Baori Medical Technology Co., Ltd. (Beijing, China). All‐In‐One 5X RT MasterMix was purchased from Aibimeng Biotechnology Co., Ltd. (Zhenjiang, China). 2×Es Taq MasterMix (Dye) was purchased from Kangwei Century Biotechnology Co., Ltd. (Taizhou, China). Bicinchoninic acid assay (BCA) protein concentration determination kit was purchased from Shanghai Biyun Tian Biotechnology Co., Ltd. (Shanghai, China).

### 4.2 Preparation of PWPI

The PWPI was prepared by referring to the method of Li et al., with slight modifications [58]. The WPI was directly dissolved in deionized water to obtain 50 mg/mL WPI stock solutions, which were stirred and hydrated overnight at 4°C (about 15 h). Then, centrifuge at 5000×g and 4°C for 10 min to remove insoluble substances. The protein content in the supernatant was determined using a BCA protein concentration determination kit before and after centrifugation, and the protein content remained unchanged. The pH of the WPI solution after centrifugation was adjusted to 4.5 using 1 mol/L HCl or 1 mol/L NaOH. Then, in a constant‐temperature magnetic stirrer (EMS‐18, Tianjin Onuo Instrument & Meter Co., Ltd., Tianjin, China), heat the WPI at 300 rpm and 90°C for 0, 5, 10, 30, and 40 min, respectively. After the heating was completed, place them in an ice bath to cool down and then obtain PWPI.

### 4.3 Characterization of PWPI

#### 4.3.1 Measurement of Particle Size and Zeta‐Potential

The particle size and zeta‐potential were measured by referring to the method of Yu et al. [59]. Samples were diluted to appropriate concentrations, and then the particle size and surface charge were detected by Malvern Zetasizer Nano‐ZS90 Dynamic Light Scattering (DLS) (Malvern Panalytical, Shanghai, China). At 25°C, the refractive index of water was 1.33, its viscosity was 0.89 mPa·s, and its dielectric constant was 78.5. The refractive index of the protein was 1.45, and the absorption rate was 0.001. The zeta‐potential was balanced for 120 s before the test, and the Smoluchowski formula was selected for fitting to obtain the zeta‐potential of WPI, PWPI, PWPI@MPNs, and PWPI@MPNs/HA. The diameters of PWPI at different heating times were analyzed using Mastersizer 3000+ Pro Laser Particle Size Analyzer (Malvern Panalytical, Shanghai, China), and the particle size reports were volume‐weighted average diameters (D4, 3).

#### 4.3.2 Turbidity

The absorbance (Abs) of WPI and PWPI samples was measured at 630 nm using an ultraviolet–visible spectrophotometer (Genesys 10S, Thermo Fisher Scientific, Waltham, MA), with deionized water as the reference for zero adjustment. Ultimately, the absorbance value was taken as the turbidity value.

#### 4.3.3 Solubility

The WPI and PWPI were centrifuged at 25°C and 10 000×g for 20 min. The protein concentrations in the supernatants before and after centrifugation were determined using the BCA kit. The formula for calculating solubility was:

$$\text{Solubility}\left(\%\right)=\frac{\text{Supernatant protein content}\;}{\text{Total protein content}}\times 100$$

#### 4.3.4 Surface Hydrophobicity

The surface hydrophobicity of the samples was determined by the 8‐Anilino‐1‐naphthalenesulfonic Acid (ANS) fluorescence probe method [60]. Dilute the protein solutions to 0, 0.2, 0.4, 0.6, 0.8, and 1 mg/mL, respectively, and then take 8 mL from each and add 90 µL of 8.0 mmol/L ANS fluorescent probe. After mixing well, react at room temperature in the dark for 15 min. The fluorescence intensity was measured using the fluorescence spectrophotometer (RF‐6000, Shimadzu, Japan) at a 3 nm bandwidth, 390 nm excitation wavelength, and 470 nm emission wavelength to calculate the hydrophobicity of the protein.

#### 4.3.5 Fluorescence Spectrum

Refer to the method of Wang et al. and Cheng et al. to measure the fluorescence spectrum, with slight modifications [19]. The WPI and PWPI were respectively diluted to appropriate concentrations, and the fluorescence intensity was recorded under the conditions of an excitation wavelength of 280 nm, a bandwidth of 3 nm, 600 nm/min, and an emission wavelength of 300 to 500 nm using the fluorescence spectrophotometer. Using the Stern‐Volmer equation to characterize the quenching mechanism:

$$\frac{F_{0}}{F}=1+K_{q}\tau_{0}C_{MPNs}=1+K_{SV}C_{MPNs}$$

*F_0_*: Fluorescence intensity before adding the quencher.

*F*: Fluorescence intensity after adding the quencher.

*K_q_*: (L·mol^−1^·s^−1^): Process constant of the quenching rate.

*C_MPNs_*: The concentration of the quenching agent (MPNs).

*K_SV_*: (L·mol^−1^): Quenching constant.

τ_0_: The average lifetime of fluorophores without quenching agents (10^−8^ s).

After transformation, the following formula was obtained:

$$\frac{1}{F}=\frac{1}{F_{0}}+\frac{K_{q}\tau_{0}}{F_{0}}\times C_{MPNs}=\frac{1}{F_{0}}+\frac{K_{SV}}{F_{0}}\times C_{MPNs}$$

The following formula was used to evaluate static quenching:

$$\mathrm{lg}\left[\frac{F_{0}-F}{F}\right]=\mathrm{lg}K_{a}+n\mathrm{lg}C_{\text{MPNs}}$$

*K_a_*: Binding constant

*n*: The number of binding sites

#### 4.3.6 Fourier Transform Infrared (FT‐IR) Spectrometer

The structure of proteins was determined using a FT‐IR spectrometer (Bruker INVENIO S FTIR, Bruker, Germany). The parameters of the instrument were set as follows: measurement range 4000–400 cm^−1^, scan time 16 times per second, and resolution 4 cm. The test procedure was to mix 1 mg of freeze‐dried protein with 150 mg of dried KBr, grind thoroughly to make the powder as fine as possible, and place it on a tablet press for tablet pressing. After preparing the samples, they were placed in the FT‐IR spectrometer for scanning, with 150 mg KBr as the blank group. Compared with the amide II band, the amide I band was more sensitive to changes in the secondary structure of proteins [61]. Therefore, the secondary structure of proteins was analyzed from 1700 to 1600 cm^−1^ through curve fitting, infrared self‐deconvolution, and peak‐splitting fitting methods. Specify 1626 cm^−1^: β‐sheet, 1641 cm^−1^: random coil, 1658 cm^−1^: α‐helix, 1676cm^−1^: β‐turn.

#### 4.3.7 Measurement Method for Surface Free Thiol Groups

Weigh 20 mg of 5,5'‐dithiobis‐(2‐nitrobenzoic acid) (DTNB) and dissolve it in 5 mL of Tris‐glycine buffer (10.4 g Tris, 6.9 g glycine, 1.2 g EDTA, make up to 1 L, and adjust pH to 8.0) to prepare Ellman reagent [19]. Take 6 mL PWPI (0.5 mg/mL) and add 20 µL of Ellman reagent. Then, incubate at room temperature for 15 min and measure the absorbance at 412 nm. The content of surface free thiol groups was calculated according to the following formula:

$$SH\left(\mu \mathrm{mol}/g\right)=\frac{73.53\times (A_{1}-A_{0})\times D}{C}$$

Among them, 73.53 was $\frac{10^{6}}{1.36\times 10^{4}}$, 10^6^ was the conversion of mg to g, and mol to mmol. 1.36 × 10^4^ was the molar absorptivity. A_1_ and A_0_ were the absorbance values of the sample and blank, respectively. C represented the concentration of the sample (mg/mL). D was the dilution factor.

### 4.4 Preparation of PWPI@MPNs/HA

MPNs were fabricated by referring to the methods of Zhu et al. and Xie et al. [13, 56]. Use Tris‐HCl buffer solution (0.01 M, pH = 8.2) to prepare 25 mL of PC solutions of 0, 0.5, 1, 2.5, 5, and 10 mg/mL, respectively, and 25 mL of Ca^2+^ solutions of 0, 0.5, 1, 2.5, 5, and 10 mg/mL, respectively. Place the PC solution in a constant‐temperature magnetic stirrer and stir at 300 rpm and 37°C. Then, slowly add the same concentration of Ca^2+^ solution and continue stirring for 10 min to form the MPNs. Add 5 mL of PWPI solution to the MPN solution and continue to stir for 1 h. Centrifuge at 8000×g, 4°C for 10 min; discard the supernatant, and wash three times with Tris‐HCl (0.01 M, pH = 8.2) to remove PC, Ca^2+^, and MPNs that were not bound to the surface of PWPI, obtaining PWPI, PWPI@MPNs5, PWPI@MPNs10, PWPI@MPNs25, PWPI@MPNs50, PWPI@MPNs100, respectively. Number 5, 10, 25, 50, and 100 here refers to the mass ratio between PWPI and MPNs (calculated by the mass of PC) for 100:5, 100:10, 100:25, 100:50, and 100:100. Resuspend the precipitate with 20 mL of 2 mg/mL HA solution, place it in a constant‐temperature magnetic stirrer, and stir at 300 rpm and 4°C for 1 h. Centrifuge at 8000×g, 4°C for 10 min, discard the supernatant, and wash the precipitate three times with deionized water to obtain PWPI, PWPI@MPNs5/HA, PWPI@MPNs10/HA, PWPI@MPNs25/HA, PWPI@MPNs50 /HA PWPI@MPNs100/HA.

### 4.5 Characterization of PWPI@MPNs/HA

#### 4.5.1 Scanning Electron Microscope (SEM)

Take a small amount of freeze–dried PWPI, PWPI@MPNs, and PWPI@MPNs/HA samples and place them on the sample stage with conductive tape attached. Then the surface of the sample was gold‐sprayed under vacuum. The shape of proteins was observed at 15 kV using SEM (SU 1510, Hitachi Corporation, Japan), and photos were taken. The diameters of the samples in SEM images of PWPI and PWPI@MPNs/HA were measured using ImageJ and statistically analyzed.

#### 4.5.2 Confocal Laser Scanning Microscope (CLSM)

Referring to the method of Fang et al. [62], Rhodamine B and FITC were used to label MPNs and HA, respectively. Then prepare PWPI@MPNs/HA using MPNs‐Rhod and HA‐FITC and observed using CLSM (LSM800, Zeiss, Germany). The excitation wavelength and emission wavelength of FITC were 491 and 516 nm, respectively. The excitation wavelength of Rhodamine B was 553nm and the emission wavelength was 610nm.

#### 4.5.3 Energy Dispersive Spectrometer (EDS)

After freeze‐drying PWPI@MPNs/HA, the distribution and content of carbon, oxygen, and calcium elements were scanned using the mapping mode of the EDS (HORIBA EX250, HORIBA, Japan).

#### 4.5.4 Isothermal Titration Calorimetry (ITC)

The binding energy of MPNs and HA was determined by ITC according to the method of Gadd et al. [63]. Before the formal determination, the MicroCal PEAQ‐ITC isothermal titration calorimeter (Malvern, UK) was treated with methanol, decon 90 lotion, and distilled water for thorough cleaning. Dissolve MPNs (1 mg/mL) and HA (1 mg/mL) respectively in Tris buffer and adjust the pH to 7. After degassing, the HA solution was added to the sample cell and balanced at 25 ± 0.01°C, while the solution was continuously stirred at 300 rpm. A total of 30 drops of MPNs solution were titrated, with 3 µL per drop. The titration interval was 120 s (ensuring that the titration peak returned to the baseline before the next titration), and the feedback mode was high. The data were fitted to the one‐site independent binding model (MicroCal PEAQ‐ITC Analyze software, Malvern, UK), obtaining the thermodynamic parameters Δ*H* (enthalpy change), Δ*S* (entropy change), *K_a_* (binding constant), and *n* (number of MPNs bound per mole of HA).

#### 4.5.5 Differential Scanning Calorimetry (DSC)

DSC analyses were obtained using a DSC calorimeter (*DSC 250, TA, USA). Briefly, 5 mg of freeze–dried powder samples PWPI and PWPI@MPNs5‐100/HA were sealed in standard aluminum trays and heated from 30°C to 160°C at a rate of 10°C/min. The sample was protected with a nitrogen atmosphere, and the flow rate of the nitrogen was 20 mL/min.

#### 4.5.6 Storage Stability

The sample PWPI@MPNs25/HA was freeze–dried and then vacuum‐packed. The material of the packaging bag is aluminum foil, with a thickness of 0.22 mm. Its oxygen transmission rate and water vapor transmission rate are 0.323 cm^3^/ (m^2^·24 h·0.1 MPa) and 0.129 g/m^2^·24 h, respectively. Then the samples were placed in two incubators with different temperatures (HWS‐30, Shaoxing Siyang Instrument Manufacturing Co., LTD, Shaoxing, China). One of them was set to 65 ± 2°C and 75 ± 5% RH, and the other to 55 ± 2°C and 75±5% RH. Take 150 mg of the sample and dissolve it in 35 mL of distilled water every three days (Days 0, 3, 6, 9, 12, etc.). Then measure the total content of PC and Ca^2+^ in the solution, as well as the content of free PC and Ca^2+^ in the supernatant after centrifugation (4°C, 10 000×g, 10 min). Calculate the proportion of free PC and Ca^2+^ in the supernatant relative to the total PC and Ca^2+^ contents. When the proportion of free PC and Ca^2+^ changes significantly compared to the 0th day, it is considered that the longest storage period at that temperature has been reached. Then calculate the storage period of the sample according to the Group Standard of China National Food Industry Association—General Guidelines for Food Shelf Life (T/CNFIA 001–2017).

$$Q_{10}=\frac{\theta_{S}\left(T_{1}\right)}{\theta_{S}\left(T_{2}\right)}$$

*Q_10_*: The ratio of storage periods at two temperatures (test temperatures *T_1_* and *T_2_*) with a temperature difference of 10°C under accelerated destructive test conditions.

$$\theta_{S}\left(T\right)=\theta_{S}\left(T^{\prime }\right)\times {Q_{10}}^{\Delta Ta/10}$$

*θ_S_* (*T*): The storage period of food at actual storage temperature T.

*θ_S_* (*T’*): The storage period obtained through accelerated destructive tests at temperature T’.

*ΔT_a_*: The difference (T’−T) between the higher temperature (T’) and the actual storage temperature (T), °C.

#### 4.5.7 Stability in an Acidic Gastric Environment

Dissolve the sample PWPI@MPNs25/HA in the simulated gastric fluid and adjust the pH to 3.0. Then, place it in the 37°C constant‐temperature water bath shaker (SHA‐B/C, Changzhou Guowang Instrument Manufacturing Co., Ltd., Changzhou, China). Shake at a speed of 180 r/min for 2 h. Then, measure the total content of PC and Ca^2+^ in the solution, as well as the content of free PC and Ca^2+^ in the supernatant after centrifugation (4°C, 10 000×g, 10 min). Calculate the proportion of free PC and Ca^2+^ in the supernatant relative to the total PC and Ca^2+^ contents.

#### 4.5.8 Stability in the Gastrointestinal Tract

After gavage of the samples PWPI@MPNs100/HA to the mice (0.84 g/kg), the mice were sacrificed and the contents of their gastrointestinal tracts were taken at 0, 1, 2, 4, 6, and 8 h. Then, dissolve the contents in 30 mL of water and measure the total content of PC and Ca^2+^ in the solution. After centrifugation (4°C, 10 000×g, 10 min), measure the content of PC and Ca^2+^ in the supernatant and calculate the proportion of free PC and Ca^2+^.

### 4.6 In Vitro Digestion

Experiments were conducted based on the in vitro digestion model used by Brodkorb et al. [64]. The components of simulated saliva fluid (SSF), simulated gastric fluid (SGF), and simulated intestinal fluid (SIF) were shown in Table 1. All electrolytes should be preheated to 37°C before use.

**TABLE 1:** Composition of in vitro digestion electrolyte.^a)^

Concentration of stock solutions			SSF (pH 7)		SGF (pH 3)		SIF (pH 7)	
	g/L	mol/L	Volume added/mL (0.5L 1 ×)	Concentration in final SSF /mmol·L^−1^	Volume added /mL (0.5L 1 ×)	Concentration in final SGF /mmol·L^−1^	Volume added /mL (0.5 L 1 ×)	Concentration in final SIF /mmol·L^−1^
KCl	37.3	0.5	15.1	15.1	6.9	6.9	6.8	6.8
KH_2_PO_4_	68.0	0.5	3.7	3.7	0.9	0.9	0.8	0.8
NaHCO_3_	84.0	1.0	6.8	13.6	12.5	25	42.5	85
NaCl	117.0	2.0	— ^b)^	—	11.8	47.2	9.6	38.4
MgCl_2_(H_2_O)_6_	30.5	0.15	0.5	0.15	0.4	0.1	1.1	0.33
(NH_4_)_2_CO_3_	48.0	0.5	0.06	0.06	0.5	0.5	—	—
HCl		6	0.09	1.1	1.3	15.6	0.7	8.4
CaCl_2_(H_2_O) _2_ ^c)^	44.1	0.3	0.025	1.5	0.005	0.15	0.04	0.6

^a)^ The table was 1 × application fluid. After adjusting the pH of the application fluid, the enzyme was directly added.

^b)^ “‐” means the corresponding salt was not added.

^c)^ CaCl_2_(H_2_O)_2_ should be added immediately before use.

Experimental process: the PWPI and PWPI@MPNs/HA samples (with PWPI concentrations of 5 mg/mL each) were mixed evenly with SSF in a 1:1 ratio (v:v), and α‐amylase (30000 U/mL) was added to make the digestion liquid reach 75 U/mL, and then placed in the 37°C constant‐temperature water bath shaker (SHA‐B/C, Changzhou Guowang Instrument Manufacturing Co., Ltd., Changzhou, China). Shake at a speed of 180 r/min for 2 min. The PWPI and PWPI@MPNs/HA samples from the oral stage were mixed with SGF in a 1:1 ratio (v:v), and then the pH of the digestive fluid was adjusted to 3.0 using 1 mol/L HCl. Subsequently, add pepsin (3000 U/mg) to make the digestive fluid reach 2000 U/mL. After adjustment, place the PWPI and PWPI@MPNs/HA sample digestion liquid in a 37°C constant‐temperature water bath shaker for shaking (180 r/min) for 2 h. After gastric digestion was completed, the digestive fluid was mixed with SIF in an equal ratio of 1:1 (v:v), and the pH was adjusted to 7.0 with 1 mol/L NaOH. Trypsin (2500 U/mg) and bile salts were added to ensure that the trypsin activity in the final small intestinal mixture was 100 U/mL and the bile salt concentration was 10 mmol/L. Then, the PWPI and PWPI@MPNs/HA sample digestion liquids were placed in a constant‐temperature water bath shaker at 37°C at a speed of 180 r/min for 2 h.

#### 4.6.1 Degree of Hydrolysis

According to the method of Yolandani et al., the degree of hydrolysis (DH) of protein after digestion was measured by OPA assay [65]. Dissolve 1.91 g of sodium tetraborate and 1 g of SDS in 70 mL of deionized water and mix well to ensure complete dissolution of the reagents. Dissolve 80 mg of OPA in 2 mL of ethanol and then add it to the above solution. After mixing, add 0.2 mL of β‐mercaptoethanol and then make up to 100 mL. The PWPI and PWPI@MPNs/HA digestion solutions were centrifuged at 10 000×g at 4°C for 10 min. 1 mL of the supernatant was taken and reacted with 3 mL of OPA reagent at room temperature for 3 min, and then the absorbance was measured at 340 nm. Prepare the 5 mmol/L L‐phenylalanine standard solution, then dilute it to 0, 1, 2, 3, 4, 5 mmol/L, and plot the standard curve. The calculation formula for the DH of the sample was:

$$DH(\%)=\frac{h}{h_{tot}}\times 100=\frac{N-N_{0}}{C\times h_{tot}}\times 100=\frac{N-N_{0}}{C}\times \frac{1}{h_{tot}}100$$

*h*: The millimoles of peptide bonds cleaved per gram of protein after hydrolysis (mmol/g)

$$h=\frac{N-N_{0}}{C}$$

*N*: Amino nitrogen concentration of PWPI digestive solution (mmol/L).

*N_0_*: Amino nitrogen concentration of protein solution (mmol/L).

*C*: Mass concentration of PWPI (g/mL).

*h_tot_*: The molar number of peptide bonds in WPI, 8.8 mmol/g.

The digestive juices of the gastric phase at 0, 30, 60, and 120 min, and the intestinal phase at 30, 60, 120, 240, and 480 min were taken, respectively, to determine the DH and turbidity.

#### 4.6.2 SDS‐PAGE Experiment

The digestion of protein was analyzed by SDS‐PAGE experiment. Take 400 µL of the digestion solution and add 100 µL of 5× protein loading buffer. Vortex to mix well and then cook in a 100°C metal bath for 10 min. The 5% concentrated gel and 12% separation gel were prepared using the SDS‐PAGE gel preparation kit. To ensure consistent protein concentration, 5 µL of the gastric phase sample and 10 µL of the intestinal phase sample were loaded. Run at 80 V for 90–120 min. The standard molecular weight of the protein marker ranged from 245 to 11 KD. Stain with Coomassie Brilliant Blue R250 staining solution for 20 min (add 1.25 g of Coomassie Brilliant Blue to 450 mL of 50% methanol solution, then add 50 mL of acetic acid), and decolorize with decolorizing solution (add 100 mL of methanol to 150 mL of acetic acid, then make up to 2 L with deionized water) until the background was clear. The gel Imager (Amersham Imager 600, GE Company, USA) was used to take photos.

#### 4.6.3 Gel Permeation Chromatography (GPC)

The distribution of molecular weight (MW) in digestive juices was analyzed using the GPC (PL‐GPC 50, Agilent Technologies, USA) [66]. The MW standard elution curve included cytochrome C (MW12384), aprotinin (MW6512), bacitracin (MW1423), Gly‐Gly‐Tyr‐Arg (MW451). Gly‐Gly‐Gly (MW189). The chromatographic column was TSK gel G2000 SWXL 300 mm × 7.8 mm. The mobile phase was acetonitrile: water: trifluoroacetic acid, with a volume ratio of 40:60:0.05. The detection wavelength was 220 nm, the flow rate was 0.5 mL/min, the column temperature was 30°C, and the injection volume was 10 µL.

#### 4.6.4 Detection of Polyphenol Content

The content of polyphenols in PWPI@MPNs/HA was determined by the Folin‐phenol reagent [19]. Prepare 1% (v:v) methanol–hydrochloric acid solution, 10% (v:v) Folin‐phenol solution, and 7.5% (w/v) sodium carbonate solution. Using PC as the standard substance, prepare a 1 mg/mL mother solution, then dilute it to 1, 0.8, 0.6, 0.4, 0.2, 0 mg/mL, and measure the standard curve. Take 0.3 mL of sample or standard solution, add 0.7 mL of hydrochloric acid methanol solution, vortex to mix well, and then add 2.5 mL of Folin‐phenol solution and 2 mL of sodium carbonate solution in sequence. After vortex mixing, incubate at 50°C for 10 min. The absorbance was measured at 760 nm, and the content of polyphenols was calculated.

#### 4.6.5 Detection of HA Content

The HA content adsorbed on the surface of PWPI@MPNs/HA was measured by the carbazole method [67]. Prepare the glucuronic acid mother solution (200 mg/L), and dilute it to 0, 10, 20, 30, 40, and 50 mg/L, respectively, to create a standard curve. Take 1 mL of the sample solution or the glucuronic acid series standard solution into a test tube with a stopper. Use 1 mL of deionized water as the blank control, add 5 mL of borax sulfuric acid solution (0.025 M), shake well, and then place it in the 100°C boiling water bath for 10 min. After that, cool it in an ice bath. The content of HA was calculated by measuring the absorbance at a wavelength of 530 nm. The content of glucuronic acid in HA was 46.43%.

### 4.7 Mouse Experiments

Specific pathogen‐free (SPF)‐grade C57BL/6J, 8‐week‐old male mice were purchased from SPF (Beijing) Biotechnology Co., Ltd. (Beijing, China), with the animal use license number: SCXK (Beijing) 2024‐0001. These experimental mice were fed in the SPF‐level breeding laboratory of China Agricultural University. The mice were placed separately in grid (stainless steel) cages, with a maximum of 6 mice in each cage. The breeding environment was a 12–12 h light and dark cycle (lights off at 18:00), with a room temperature of 22 ± 1°C and a relative humidity of 55±15%. Before the experiment began, the mice adapted to the facility for 7 days. During this period, they could consume AIN‐93G and drink water ad libitum. All mouse experiments were conducted strictly in accordance with Beijing Laboratory Animal Management. The experiment was approved by the Institutional Animal Care and Use Committee of China Agricultural University. The approval number was AW13305202‐5‐1.

#### 4.7.1 Changes in Serum AAs Concentration After Gavage

After 7 days of adaptive feeding, mice were randomly divided into 6 groups, with n = 18 in each group, namely (1) PWPI group, (2) PWPI@MPNs5/HA group, (3) PWPI@MPNs10/HA group, (4) PWPI@MPNs25/HA group, (5) PWPI@MPNs50/HA group, and (6) PWPI@MPNs100/HA group. The mice were fasted overnight for 16 h before the experiment, during which time they were free to drink water. The PWPI content of all groups administered by gavage was 0.84 g/kg. After gavage, blood was collected from the orbit at 0, 1, 2, 4, 6, and 8 h, respectively, and placed in the separation gel accelerator tube. The amounts of casein and cross‐linked whey protein administered by gavage were both 0.84 g/kg. These two groups were named CAS and CWPI, respectively. CWPI was cross‐linked using the transglutaminase enzyme (TGase). Specifically, after adjusting the pH of the whey protein solution to 6.0, add TGase at a rate of 2% (m/m) of whey protein. Then react in a water bath at 50°C for 2 h. After the reaction was completed, heat at 90°C for 10 min to inactivate the TGase. After gavage of CAS and CWPI, blood was collected from the orbit at 0, 1, 2, 3, 4, and 6 h, respectively. In addition, another experiment was conducted in which the same amount of whey protein was evenly divided into six gavage doses. This group was named EWPI. Specifically, at 0, 1, 2, 3, 4, and 5 h, a dosage of 0.14 g/kg was administered by gavage, respectively. Blood was collected from the orbit at 0, 1, 2, 4, 6, and 8 h, respectively. After placing the plasma at room temperature for 2–3 h, centrifuge it at 3500×g for 15 min, collect the supernatant, and measure the concentration of serum AAs.

#### 4.7.2 Determination of Serum AAs

The concentration of serum AAs was measured by referring to the method of Song et al. [68]. Take 50 µL of serum and add 350 µL of pre‐cooled methanol for protein precipitation. Then, centrifuge for 10 min at 4°C and 13 000×g, and take the supernatant for vacuum freeze‐drying. Then, the AAs were reconstituted in an equal ratio of acetonitrile to water (85:15, v:v). After reconstitution, centrifuge at 13,000×g for 5 min at 4°C and take 100 µL of the supernatant into the mass spectrometry injection bottle. Prepare a 200 ppm mixed standard solution, then dilute it several times in a gradient to obtain the AAs standard solution. Measure them together with the sample on the machine and draw a standard curve.

The concentration of serum AAs was measured by combining a high‐performance liquid chromatography (HPLC) system (LC‐40D X3, Shimadzu Corporation, Japan) with a quadrupole time‐of‐flight mass spectrometer (TripleToF 7600, AB SCIEX Corporation, USA). The liquid chromatography column was BEN Admine (2.1 mm×100 mm, 1.7 µL) and was equipped with an Admine guard column (2.1 mm ×10 mm, 1.7 µL). The chromatographic separation temperature was 45°C. Mobile phase A was an aqueous solution containing 20 mmol/L ammonium acetate and 0.5% formic acid, and mobile phase B was an acetonitrile: water (85:15, v:v) solution containing 20 mmol/L ammonium acetate and 0.5% formic acid. The operating conditions of the machine were gradient elution at a mobile phase velocity of 0.3 mL/min, with 15% B phase for 0–10.0 min, 100% B phase for 10.0–14.0 min, 100%→15% B phase for 14.0–14.1 min, and 15% B phase for 14.1–17 min. AAs data collection adopted a multi‐reaction monitoring mode, including ion scanning parameters for 20 kinds of AAs. All AAs data collection and analysis were carried out on SCIEX OS instruments. The working parameters of the instrument were set as follows: the source temperature was 550°C; both ion source gas 1 and ion source gas 2 were 55 psi, the curtain gas was 35 psi, the ion spray voltage was 5500 V, and the de‐clustering voltage was 50 V. During the automatic MS/MS acquisition, the instrument's scanning parameters were set to a mass‐to‐charge ratio range of 60–1000 Da, with an accumulation time of 100 ms.

#### 4.7.3 Fluorescence Imaging of Small Animals

Fluorescence imaging was measured by referring to the method of Bao et al. [69]. After preparing the PWPI solution, adjust the pH value to 8.5 ± 0.5. Dilute Cy5 to 10 mg/mL using DMSO, add Cy5 solution at a ratio of 1 mg Cy5: 100 mg PWPI, and then place it in an ice bath on a shaker for 4 h to complete the labeling of PWPI. Place the PWPI solution in a 10 KDa ultrafiltration tube, centrifuge at 4°C and 4000×g for 20 min to remove the free Cy5 dye, add PBS to resuspend the PWPI, and repeat ultrafiltration 5 times to obtain PWPI‐Cy5. Prepare PWPI‐Cy5@MPNs25/HA using PWPI‐Cy5. After 7 days of adaptive feeding, the mice were randomly divided into 2 groups, with 6 mice in each group. On the day before the experiment, the mice were fasted overnight for 16 h. Each mouse was administered PWPI‐Cy5 and PWPI‐Cy5@MPNs25/HA 0.84 g/kg, respectively (PWPI‐Cy5@MPNs25/HA was calculated by the weight of PWPI in the sample). The mice were sacrificed at 0, 1, 2, 4, 6, and 8 h after gavage, respectively. The gastrointestinal tracts were dissected and placed in a small animal fluorescence imaging system (IVIS Lumina LT, Revvity, Massachusetts, USA) to observe the fluorescence signals and take photos for analysis.

### 4.8 Establishment of Sarcopenia Models and Nutritional Intervention

After 7 days of adaptation, the mice were randomly divided into 9 groups, with n = 6 in each group, namely (1) control group (Con), (2) dexamethasone group (Dex), (3) dexamethasone+PWPI group (Dex+PWPI), (4) dexamethasone+PWPI@MPNs5/HA group (Dex+PWPI@MPNs5/HA). (5) dexamethasone+PWPI@MPNs10/HA group (Dex+PWPI@MPNs10/HA), (6) dexamethasone+PWPI@MPNs25/HA group (Dex+PWPI@MPNs25/HA), (7) dexamethasone+PWPI@MPNs50/HA group (Dex+PWPI@MPNs50/HA), (8) dexamethasone+PWPI@MPNs100/HA group (Dex+PWPI@MPNs100/HA), and (9) dexamethasone+ MPNs/HA group (Dex+MPNs/HA). All Dex models were made by intraperitoneal injection of dexamethasone at a dose of 24 mg/kg/d [53]. The gavage dose of PWPI and Dex+PWPI@MPNs5‐100/HA was 0.84 g/kg/d (calculated by the mass of PWPI). The doses of MPNs/HA in the Dex+MPNs/HA group were the same as those in the Dex+PWPI@MPNs25/HA group. The doses were 0.16 g/kg/d for MPNs and 0.11 g/kg/d for HA. The Con group used PBS instead of the samples and dexamethasone. The method of sarcopenia models and nutritional intervention simultaneously was adopted, and the experimental period was 14 days. During this period, the food intake and body weight of the mice were recorded. After the end of the experimental period, the muscle‐related behavior of the mice was measured. Then, the mice were sacrificed, and the gastrocnemius muscle (GA), tibialis anterior muscle (TA), soleus muscle (SOL), and quadriceps femoris (QA) muscle of their hindlimbs were collected. Photos were taken, and their weights were measured to calculate the skeletal muscle index. Parts of the GA and TA were placed in 4% paraformaldehyde solution for histopathological research, while the other part was frozen in liquid nitrogen and stored at −80°C for subsequent molecular experiments.

#### 4.8.1 Body Composition Measurement

Referring to the method of Wu et al. [70], body composition measurement was performed using the body composition analysis and imaging system of awake small animals (MesoQMR23‐060H‐I, Shanghai Nuomai Electronic Technology Co., Ltd., Shanghai, China). Before the test, the mice were weighed and then placed in separate instruments. After the measurement was completed, the physiological parameters such as lean meat and fat content of the mice in the awake state were obtained. The calculation formula was as follows:

$$\text{Fat content}\left(\%\right)=\frac{\text{Fat weight}}{\text{Body weight}}\times 100\%$$

$$\text{Lean meat content}\;\left(\%\right)=\frac{\text{Lean meat weight}}{\text{Body weight}}\times 100\%$$

#### 4.8.2 Inverted Grid Test

Place a 40 cm × 40 cm metal grid (with a diameter of 2 mm and each small grid side length of 20 mm) on a frame at a vertical height of 60 cm from the horizontal ground. Place a 5 cm soft pad directly below the grid, so that the distance between the metal grid and the soft pad was 55 cm. During the test, first place the mouse at the center of the grid, then gently turn the grid upside down so that the mouse's body was facing down and in a suspended state. The suspension period started from the inverted grid and lasted until the mouse fell. Each mouse was tested three times, with an interval of ≥30 min between each test. The average of the three times as the final result. If the mouse drops within 10 s, the test should be repeated immediately to ensure the accuracy of the result. The test was based on the mice's instinctive fear of falling. It was best to have the mice complete the test with as few repetitions as possible in an unknown state. The scoring criterion was the hanging time (in seconds) divided by 100 to obtain the score. For example, 180 s would be counted as 1.8 points, with a full score of 10 points. Therefore, a score of 10 points was for ≥1000 s.

#### 4.8.3 Grip Strength Test

The grip strength of mice was measured with reference to Yu et al. [53]. The grip strength of the forelimbs and hindlimbs of mice was tested using the mouse and rat grip strength tester (ZS‐ZL, Beijing Zhongshi Dichuang Technology Development Co., Ltd., Beijing, China). First, place the instrument horizontally, turn on the sensor, and select the peak mode. Then place the mouse on the grid sensing platform of the instrument. When the mouse's front or hind limbs grasp the grid, gently pull the mouse's tail horizontally until it can no longer hold the grip and detach from the grid platform. Throughout the entire process, the mouse's body was kept in a horizontal position, and the speed of pulling the mouse was maintained at a constant rate. The instrument would record the maximum grip force of the mouse throughout the entire process, and the force applied to the instrument was far from reaching the maximum intensity it could withstand. Each mouse was measured three times, and the average value was taken as the final result.

#### 4.8.4 Treadmill Exhaustion Test

The muscle endurance of mice was measured using a small animal treadmill (ZS‐PT‐III, Beijing Zhongshi Dichuang Technology Development Co., Ltd., Beijing, China) [70]. Two days before the formal test, the mice were subjected to adaptive training according to the parameters in Table 2. Once a day, for 5 min each time.

**TABLE 2:** Parameters of the treadmill during the adaptation stage.

	Speed (m/min)	Acceleration time (s)	Velocity duration (min)
Initial velocity	12	5	4
First‐order velocity	16	5	4
Second‐order velocity	20	5	2

During the formal test, the parameters in Table 3 were adopted to set up the treadmill. The stimulation current set for this stage was 0.5mA. When the mice stopped running, they were stimulated with current and noise. If the mice still did not stop running after 10 s, it was considered that they were exhausted. The time and distance of exhaustion were recorded.

**TABLE 3:** Parameters of the treadmill during the formal measurement stage.

	Speed (m/min)	Acceleration time (s)	Velocity duration (min)
Initial velocity	12	5	4
First‐order velocity	16	5	4
Second‐order velocity	20	5	2

#### 4.8.5 Paraffin Section Preparation of Muscle Tissue and HE Staining

The GA and TA were placed in an embedding cage lined with embedding paper, and then fixed in 4% paraformaldehyde for 24 h. Then, dehydration, paraffin embedding, and dewaxing were carried out by referring to the methods of Grover et al. [71]. Add hematoxylin dye to the GA and TA muscle tissues, stain at room temperature for 6 min, then add tap water for bluing for 5 min, and then stop bluing in deionized water. Place the sections in 95% ethanol for 30 s, eosin dye for 30 s, 95% ethanol for 30 s, anhydrous ethanol I for 1 min, anhydrous ethanol II for 1 min, xylene I for 10 min, and xylene II for 10 min. Add neutral resin to the microscope slide and cover it with a coverslip. Then place it in a fume hood for more than 48 h and observe and photograph it under a microscope.

#### 4.8.6 Western Blot (WB)

The proteins in the GA were extracted by referring to the method of Hosoi et al. [72]. Measure the protein concentration using the BCA kit and dilute the supernatant to between 1 and 2 µg/µL. Then, the supernatant was mixed with 5×loading buffer at a ratio of 1:4, heated in a 100°C metal bath for 10 min, and stored at −20°C for WB detection. Prepare 5% concentrated gel and 10% separated gel using the SDS‐PAGE gel preparation kit. The extracted proteins and markers were added at a rate of 10–30 µL per well, and the proteins were separated under a constant pressure of 80 V for 100–120 min. The proteins were transferred to a polyvinylidene fluoride (PVDF) membrane for 2 h under the conditions of a constant current of 200 mA in an ice bath. Take out the PVDF membrane and seal it at room temperature with rapid sealing solution on a shaker for 15 min. Take out the PVDF membrane and pour in the primary antibody (dilute according to the instructions). The name, brand, and product number were shown in Table 4. Incubate overnight at 4°C. Then, dilute the secondary antibody with rapid blocking solution at a ratio of 1:5000 and incubate it at room temperature on a shaker for 1 h. ECL hypersensitive luminescence solutions A and B were mixed in a 1:1 ratio, dropped onto the PVDF membrane, and exposed and photographed using a gel Imager (Amersham Imager 600, GE Company, USA).

**TABLE 4:** The name, brand, and product number of the primary antibody.

Antibody name	Brand	Product number
Phospho‐Akt (Thr308) (244F9) Rabbit Monoclonal Antibody	Cell Signaling Technology	Catalog# 4056S
AKT1/2/3 Recombinant Rabbit Monoclonal Antibody	HuaBio	Catalog# HA721870
Phospho‐mTOR (S2448) Mouse Monoclonal Antibody	HuaBio	Catalog# HA600094
mTOR rabbit monoclonal antibody	Cell signaling technology	Catalog# 2972S
Phospho‐p70 S6 Kinase (S434) Mouse Monoclonal Antibody	HuaBio	Catalog# RT1456
p70 S6 Kinase Recombinant Rabbit Monoclonal Antibody	HuaBio	Catalog# HA722520
Phospho‐4E‐BP1/2/3 (T45) Mouse Monoclonal Antibody	HuaBio	Catalog# RT1004
eIF4EBP1 Recombinant Rabbit Monoclonal Antibody	HuaBio	Catalog# ET1701‐83
eIF4G Rabbit Polyclonal Antibody	HuaBio	Catalog# ER63337
MAFbx mouse monoclonal antibody	Santa Cruz	Catalog#sc‐166806
MuRF1 mouse monoclonal antibody	Santa Cruz	Catalog#sc‐398608
β‐Actin (D6A8) Rabbit Monoclonal Antibody #8457	Cell Signaling Technology	Catalog#8457S

#### 4.8.7 Reverse Transcription‐Polymerase Chain Reaction (RT‐PCR)

The key genes were quantified by referring to the method of Cirak et al. [73]. After extracting RNA from the GA muscle, reverse transcription was performed using a gene amplification instrument (T100, Bio‐Rad, USA). The reaction system was added in the dark in the special eight‐row 200 µL centrifuge tubes for RT‐PCR: 10 µL *TB Green Premix Ex Taq enzyme*, 0.5 µL upstream primers, 0.5 µL downstream primers, 3.6 µL DEPC water, 0.4 µL fluorescent dye, and 5 µL cDNA diluted with DEPC water. The primer design was shown in Table 5. Quantification was performed using a QuantStudioTM 5 Real‐time PCR instrument (Thermo Fisher Scientific, USA). The program was set at 95°C for 30 s, 40 cycles at 95°C for 5 s, 50–70°C (set according to the T_m_ value of the primers) for 30 s, and 4°C ∞. Taking β‐actin as the internal reference, the mRNA expression level of the target gene was calculated by the 2^−△△CT^ method.

**TABLE 5:** Primers for RT‐PCR in this study.

Gene name	Forward primer (5′ to 3′ direction)	Reverse primer (5′ to 3′ direction)
*PI3K*	GCCAGTGGTCATTTGTGTTG	ACACAACCAGGGAAGTCCAG
*Akt1*	ATGAACGACGTAGCCATTGTG	TTGTAGCCAATAAAGGTGCCAT
*mTOR*	TCCTGCGCAAGATGCTCATC	TGTGCTCCAGCTCTGTCAGGA
*Foxo3*	CTAGAATTTCGTGTGGCGCG	TGAGAAGAAAGGCGGCAGAG
*Atrogin‐1*	CAGCTTCGTGAGCGACCTC	GGCAGTCGAGAAGTCCAGTC
*MuRF‐1*	GACAGTCGCATTTCAAAGCA	GCCTAGCACTGACCTGGAAG
*β‐actin*	GGACTGTTACTGAGCTGCGTT	CGCCTTCACCGTTCCAGTT

### 4.9 Statistical Analysis

At least three independent experiments were performed, and all data were shown as mean ± standard error. The differences between the mean values were analyzed using one‐way ANOVA post hoc Duncan's multiple range test (*p* < 0.05) with software SPSS 26.0 (SPSS Inc., Chicago, IL, USA).

## Author Contributions

**Yafei Zhang**: writing – original draft, writing – review and editing. **Yuning Zhang**: methodology. **Yongjian Ai**: software, visualization. **Yao Lu**: data curation. **Heng Quan**: methodology. **Zhen Zhang**: investigation. **Yuqi Wang**: formal analysis. **Yujia Luo**: validation. **Xiaoxu Zhang**: methodology. **Yingying Lin**: validation. **Sijia Song**: conceptualization, project administration. **Huiyuan Guo**: conceptualization, supervision, funding acquisition.

## Conflicts of Interest

We had full access to all of the data in this study, and we take complete responsibility for the integrity of the data and the accuracy of the data analysis. In addition, we declare that we have no conflicts of interest in this work. We declare that we do not have any commercial or associative interest that represents a conflict of interest in connection with the work submitted.

## Supporting information




**Supporting File**: advs77951‐sup‐0001‐SuppMat.docx.

## Data Availability

The data that support the findings of this study are available from the corresponding author upon reasonable request.
