# Supplementary material for: Developing Metal–Polyphenol Network‐Based Single‐Protein Particle Encapsulation System for Sustained Release of Whey Protein to Mitigate Sarcopenia
Source: Adv Sci (Weinh). 2026 Sep 27:e77951. Online ahead of print. doi: 10.1002/advs.77951 (PMC13616254; doi:10.1002/advs.77951)
Supplement: Supplementary file 1 [file ADVS-9999-e77951-s001.docx]

Supporting Information

**Developing** **Metal-Polyphenol Network-Based Single-Protein Particle Encapsulation System for** **Encapsulated and Sustained Release of Whey Protein to Mitigate Sarcopenia**

*Yafei Zhang, Yuning Zhang, Yongjian Ai, Yao Lu, Heng Quan, Zhen Zhang, Yuqi Wang, Yujia Luo, Xiaoxu Zhang, Yingying Lin, Sijia Song*, Huiyuan Guo**

Y.F. Zhang, Y.N. Zhang, Y. Lu, Z. Zhang, Y.Q. Wang, H.Y. Guo

College of Food Science and Nutritional Engineering,

China Agricultural University, Beijing 100083, China

E-mail: guohuiyuan@cau.edu.cn (H.Y. Guo)

Y.J. Ai, H. Quan, Y.J Luo, X.X. Zhang, Y.Y. Lin, S.J. Song, H.Y. Guo

MOE Key Laboratory of Precision Nutrition and Food Quality,

Department of Nutrition and Health,

China Agricultural University, Beijing 100083, China

E-mail: songsijia1996@cau.edu.cn (S.J. Song)

**Figure S1** Isoelectric point measurement of WPI. Mean ± SE of n=3 (at least) independent experiments.

**Figure S2** Particle size changes of WPI during heating process. Mean ± SE of n=3 (at least) independent experiments. Different lowercase letters indicated significant differences within groups (*p* < 0.05).

**Figure S3** The turbidity changes of WPI during the heating process. Mean ± SE of n=3 (at least) independent experiments. Different lowercase letters indicated significant differences within groups (*p* < 0.05).

**Figure S4** The changes in solubility of WPI during the heating process. Mean ± SE of n=3 (at least) independent experiments. Different lowercase letters indicated significant differences within groups (*p* < 0.05).

**Figure S5** Digital image of the solubility change of WPI during the heating process.

**Figure S6** The change of surface hydrophobicity of WPI during the heating process. Mean ± SE of n=3 (at least) independent experiments. Different lowercase letters indicated significant differences within groups (*p* < 0.05).

**Figure S7** The changes of free thiol groups on the surface of WPI during the heating process. Mean ± SE of n=3 (at least) independent experiments. Different lowercase letters indicated significant differences within groups (*p* < 0.05).

**Figure S8** The changes in fluorescence intensity of WPI during the heating process.

**Table S1** The changes in the maximum fluorescence intensity (a.u._max_) and the maximum fluorescence emission wavelength (λ_max_) of WPI during the heating process.

|  | Native protein | pH4.5 | 5 min | 10 min | 20 min | 30 min | 40 min |
| --- | --- | --- | --- | --- | --- | --- | --- |
| a.u._max_ | 1856.3±25.7^e^ | 2221.2±21.1^d^ | 2972.9±35.5^c^ | 3781.0±77.5^b^ | 4075.0±76.3^a^ | 4121.8±86.4^a^ | 4122.2±59.6^a^ |
| λ_max_(nm) | 326.0±1.0^c^ | 325.3±1.5^c^ | 328.3±1.5^b^ | 330.3±0.6^a^ | 331.7±1.2^a^ | 330.7±0.6^a^ | 331.3±0.6^a^ |

Mean ± SE of n=3 (at least) independent experiments. Different lowercase letters indicated significant differences within groups (*p* < 0.05).

**Figure S9** The FT-IR spectrum of WPI after heating at different times.

**Table S2** The changes of β-sheet, α-helix, β-turn and Random coil of WPI during the heating process.

|  | β-sheet (%) | α-helix (%) | β-turn (%) | Random coil (%) |
| --- | --- | --- | --- | --- |
| 0 min | 35.42±1.08^a^ | 23.15±1.44^a^ | 22.99±0.81^a^ | 18.44±0.72^d^ |
| 5 min | 31.93±1.28^b^ | 23.19±1.63^a^ | 20.26±1.28^b^ | 24.63±1.66^c^ |
| 10 min | 26.89±1.33^c^ | 25.04±1.42^a^ | 18.17 ±0.63^c^ | 29.90±0.30^b^ |
| 20 min | 25.29±1.14^cd^ | 24.01±0.47^a^ | 17.61±0.77^c^ | 33.09±0.16^a^ |
| 30 min | 24.63±1.13^d^ | 23.74±0.52^a^ | 18.44±1.41^c^ | 33.19±0.49^a^ |
| 40 min | 26.07±1.09^cd^ | 22.59±2.71^a^ | 18.22±0.99^c^ | 33.13±0.86^a^ |

Mean ± SE of n=3 (at least) independent experiments. Different lowercase letters indicated significant differences within groups (*p* < 0.05).

**Table S3** The maximum fluorescence intensity (a.u._max_), maximum fluorescence emission wavelength (λ_max_), and fluorescence quenching efficiency in the fluorescence spectrum of WPI after binding with different amounts of MPNs.

|  | PWPI | PWPI@MPNs5 | PWPI@MPNs10 | PWPI@MPNs25 | PWPI@MPNs50 | PWPI@MPNs100 |
| --- | --- | --- | --- | --- | --- | --- |
| a.u._max_ | 4319.7±75.3^a^ | 1872.1±49.4^b^ | 856.4±23.4^c^ | 133.3±6.9^d^ | 22.8±2.9^e^ | 8.9±1.2^e^ |
| λ_max_(nm) | 331.7±1.2^c^ | 330.7±0.6^c^ | 331.7±1.5^c^ | 329.7±0.6^c^ | 345.3±13.4^b^ | 393.0±11.5^a^ |
| Quenching efficiency (%) | 0.00 | 56.67±0.47^d^ | 80.17±0.65^c^ | 96.91±0.16^b^ | 99.47±0.06^a^ | 99.79±0.03^a^ |

Mean ± SE of n=3 (at least) independent experiments. Different lowercase letters indicated significant differences within groups (*p* < 0.05).

**Figure S10** A) Digital image of the sample PWPI@MPNs25/HA after freeze-drying; B) Digital image of the sample PWPI@MPNs25/HA after vacuum packaging.

**Figure S11** During the storage period of 0-12 d at 65℃, the changes in A) the proportion of free PC, B) the proportion of free calcium ions, C) and particle size after the sample PWPI@MPNs25/HA was dissolved. Mean ± SE of n=3 (at least) independent experiments. Different lowercase letters indicated significant differences within groups (*p* < 0.05). *NS* indicated no significant differences within groups (*p* > 0.05).

**Figure S12** During the storage period of 0-30 d at 55℃, the changes in A) the proportion of free PC, B) the proportion of free calcium ions, C) and particle size after the sample PWPI@MPNs25/HA was dissolved. Mean ± SE of n=3 (at least) independent experiments. Different lowercase letters indicated significant differences within groups (*p* < 0.05). *NS* indicated no significant differences within groups (*p* > 0.05).

**Figure S13** The comparison of A) free PC and B) free calcium ions in PWPI@MPNs25/HA after incubation in acidic SGF with pH 3 for 2 h. Mean ± SE of n=3 (at least) independent experiments. *NS* indicated no significant differences within groups (*p* > 0.05).

**Figure S14** The recovery rate of PC and the proportion of free PC after gavage. Mean ± SE of n=3 (at least) independent experiments. Different lowercase letters indicated significant differences within groups (*p* < 0.05). *NS* indicated no significant differences within groups (*p* > 0.05).

**Figure S15** The recovery rate of calcium ion and the proportion of free calcium ion after gavage. Mean ± SE of n=3 (at least) independent experiments. Different lowercase letters indicated significant differences within groups (*p* < 0.05). *NS* indicated no significant differences within groups (*p* > 0.05).

**Figure S16** Digital images of digestive juices of PWPI and PWPI@MPNs5/HA-PWPI@MPNs100/HA in the gastric phase at 0-120 min and intestinal phase at 0-480 min during *in vitro* digestion.

**Figure S17** The digestion status of PWPI and PWPI@MPNs5/HA-PWPI@MPNs100/HA during *in vitro* digestion, including the changes in DH during gastric digestion (0-120 min) and intestinal digestion (0-480 min) stages.

**Figure S18** The turbidity changes of digestive juices in the gastric phase 0-120 min and the intestinal phase 0-480 min during *in vitro* digestion with A) PWPI and B-F) PWPI@MPNs5/HA-PWPI@MPNs100/HA. Mean ± SE of n=3 (at least) independent experiments. Different lowercase letters indicated significant differences within groups (*p* < 0.05).

**Table S4** The distribution of MW in the digestive juices after *in vitro* digestion of PWPI and PWPI@MPNs5/HA-PWPI@MPNs100/HA.

|  | PWPI | | PWPI@MPNs5/HA | | PWPI@MPNs10/HA | | PWPI@MPNs25/HA | | PWPI@MPNs50/HA | | PWPI@MPNs100/HA | |
| --- | --- | --- | --- | --- | --- | --- | --- | --- | --- | --- | --- | --- |
| MW distribution | % | Mw | % | Mw | % | Mw | % | Mw | % | Mw | % | Mw |
| >10000 | 1.52 | 13504 | 1.49 | 13947 | 1.38 | 13213 | 1.18 | 13729 | 1.37 | 15262 | 1.64 | 13831 |
| 10000~5000 | 0.73 | 6999 | 1.15 | 6887 | 1.12 | 6865 | 1.82 | 6765 | 2.18 | 6761 | 4.00 | 6872 |
| 5000~3000 | 1.33 | 3766 | 1.99 | 3793 | 2.06 | 3788 | 3.13 | 3808 | 3.92 | 3783 | 5.53 | 3819 |
| 3000~2000 | 2.69 | 2401 | 3.68 | 2407 | 3.42 | 2409 | 5.21 | 2414 | 5.77 | 2439 | 7.43 | 2438 |
| 2000～1000 | 10.88 | 1422 | 11.28 | 1449 | 11.51 | 1438 | 12.84 | 1452 | 13.30 | 1406 | 14.36 | 1436 |
| 1000~500 | 28.97 | 675 | 29.06 | 680 | 29.84 | 676 | 31.12 | 685 | 34.05 | 697 | 30.03 | 703 |
| 500～180 | 41.91 | 319 | 40.41 | 321 | 39.55 | 323 | 34.92 | 327 | 28.54 | 338 | 27.38 | 331 |
| ＜180 | 11.98 | 118 | 10.94 | 114 | 11.12 | 110 | 9.78 | 107 | 10.88 | 101 | 9.63 | 100 |

**Figure S19** Particle size of CWPI, A) Particle size distribution of CWPI and B) Comparison of particle sizes between CWPI and WPI. Mean ± SE of n=3 (at least) independent experiments. The “**” note as very significant difference with *p* < 0.01.

**Figure S20** Heatmap of changes in serum AAs after gavage of A) CAS and B) CWPI; C) The changes of total serum AAs during the period of 0-8 h after gavage of CAS and CWPI, respectively. Mean ± SE of n=3 (at least) independent experiments.

**Figure S21** A) Heatmap of changes in serum AAs after gavage of EWPI; B) The changes of total serum AAs during the period of 0-8 h after gavage of EWPI and PWPI@MPNs25/HA, respectively. Mean ± SE of n=3 (at least) independent experiments.

**Figure S22** The total radiant efficiency of fluorescence imaging after gavage of A) PWPI-Cy5 and B) PWPI-Cy5@MPNs25/HA. Mean ± SE of n=3 (at least) independent experiments. Different lowercase letters indicated significant differences within groups (*p* < 0.05).

**Figure S23** The weight of A) lean meat and B) fat after MPNs/HA treatment. Mean ± SE of n=6 (at least) independent experiments. Different lowercase letters indicated significant differences within groups (*p* < 0.05).

**Figure S24** The particle size of WPI.

**Figure S25** The particle size of WPI after heating at different times (0-120 min). Mean ± SE of n=3 (at least) independent experiments. Different lowercase letters indicated significant differences within groups (*p* < 0.05).

**Figure S26** The MPNs are the structure formed by polyphenols through *ortho*-dihydroxyl groups (A), 5-hydroxyl groups and/or 3-hydroxyl groups, and ketone groups of C4 (B-C). D) Schematic diagram of polyphenols bonding with the peptide groups of the protein molecular skeleton through double H-bonds with their *ortho*-phenolic hydroxyl groups. The hydrogen atom in one of the phenolic hydroxyl groups acted as the H donor, and the carbonyl oxygen in the peptide groups acted as the H acceptor.

**Figure S27** A) Schematic diagram of the structure formed by PWPI and different thicknesses of shell MPNs/HA. B) Schematic diagram of the contact form between digestive enzymes and PWPI, PWPI@MPNs5-100/HA during digestion. The more shell MPNs/HA was used, the smaller the exposed surface area of PWPI that comes into contact with digestive enzymes, and the slower the digestion rate. When the MPNs/HA shell completely covers the PWPI, the PWPI basically does not come into contact with digestive enzymes and thus is hardly digested.

**Figure S28** A) Scheme design for sarcopenia model establishment and MPNs/HA treatment; B) Weight changes during the sarcopenia model establishment period; C) Food intake changes in sarcopenia model establishment period; D) lean body content, E) fat body content, F) lean weight, G) fat weight, H) forelimb grip strength, I) hindlimb grip strength, J) grid hanging time score, K) exhaustion time, and L) exhaustion distance of the mice were measured after 14 days of intraperitoneal Dex injection and treatment. Mean ± SE of n=6 (at least) independent experiments. Different lowercase letters indicated significant differences within groups (*p* < 0.05).

**Figure S29** A) The digital images of TA, GA, SOL and QA of the hindlimbs treated with MPNs/HA; The muscle index of B) TA, C) GA, D) SOL, and E) QA; F) HE staining images and the CSA statistical analysis results of GA; G) HE staining images and the CSA statistical analysis results of TA. Mean ± SE of n=6 (at least) independent experiments. Different lowercase letters indicated significant differences within groups (*p* < 0.05).
